# HBO‐PC Reprograms Neuroimmune Metabolism Through Disruption of the LRG1‐HIF‐1α‐IL‐6‐STAT3 Amplification Loop Attenuates Pyroptosis and Ischemia–Reperfusion Injury

**DOI:** 10.1002/cns.70907

**Published:** 2026-04-29

**Authors:** Wenying Lv, Junzhe Lv, Kexin Xiong, Shunshun Yuan, Jingyao Yang, Dazhi Guo

**Affiliations:** ^1^ Department of Hyperbaric Oxygen The Sixth Medical Center, PLA General Hospital Beijing China; ^2^ The Second School of Clinical Medicine Southern Medical University Guangzhou China; ^3^ Medical School of Chinese PLA Beijing China; ^4^ School of Medicine South China University of Technology Guangzhou China

**Keywords:** CIRI, GSDME, HBO‐PC, LRG1, pyroptosis

## Abstract

**Background:**

Neuroinflammation and pyroptosis driven by excessive microglial activation play key roles in cerebral ischemia–reperfusion injury (CIRI). Hyperbaric oxygen preconditioning (HBO‐PC) exhibits neuroprotective effects, but its mechanisms remain unclear. Leucine‐rich α‐2‐glycoprotein 1 (LRG1) is implicated in CIRI pathology, yet whether HBO‐PC modulates neuroinflammation and pyroptosis via LRG1 is unknown.

**Methods:**

A mouse CIRI model was generated by middle cerebral artery occlusion. HBO‐PC and LRG1 siRNA knockdown were applied. Neurological function and molecular changes were evaluated.

**Results:**

HBO‐PC reduced infarct volume and improved neurological outcomes, while downregulating LRG1 in neurons and microglia. LRG1 promoted HIF‐1α accumulation by inhibiting prolyl hydroxylase activity, leading to caspase‐3‐dependent GSDME cleavage and pyroptosis. Released IL‐6 activated STAT3, which further transcriptionally upregulated LRG1, forming a self‐amplifying HIF‐1α/GSDME/IL‐6/STAT3 loop. HBO‐PC disrupted this circuit and shifted microglia toward an anti‐inflammatory phenotype.

**Conclusion:**

HBO‐PC protects against CIRI by breaking the LRG1–HIF‐1α–IL‐6–STAT3 feedback loop, thereby attenuating pyroptosis and neuroinflammation while promoting anti‐inflammatory microglial polarization. LRG1 represents a promising therapeutic target in ischemic brain injury.

## Graphical abstract

A mouse CIRI model was generated by middle cerebral artery occlusion. HBO‐PC and LRG1 siRNA knockdown were applied. Neurological function and molecular changes were evaluated. We confirmed that HBO‐PC reprograms neuroimmune metabolism through disruption of the LRG1‐HIF‐1α‐IL‐6‐STAT3 amplification Loop attenuates pyroptosis and ischemia–reperfusion injury.

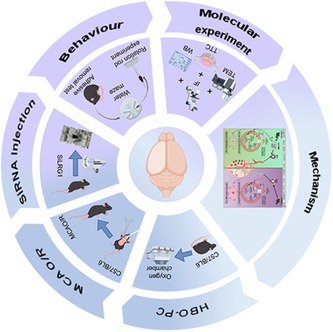

**Abbreviations**
ATA: atmospheres absolute
BBB: blood–brain barrier
CCA: common carotid artery
CIRI: cerebral ischemia–reperfusion injury
ECA: external carotid artery
GSDMD: gasdermin D
GSDME: gasdermin E
HBO‐PC: Hyperbaric oxygen preconditioning
HIF‐1: hypoxia‐inducible factor 1
ICA: internal carotid artery
IL‐6R: IL‐6 receptor
LRG1: Leucine‐rich α‐2 glycoprotein 1
LRG1: Leucine‐rich α‐2‐glycoprotein 1
MCAO/R: middle cerebral artery occlusion/reperfusion
MMP‐9: matrix metalloproteinase‐9
mNSS: modified Neurological Severity Score
MWM: Morris water maze
PBS: phosphate‐buffered saline
PFA: paraformaldehyde
PHDs: prolyl hydroxylases
ROS: reactive oxygen species
TTC: 2,3,5‐triphenyltetrazolium chloride

## 1 Introduction

The data from the American Stroke Association indicates that stroke remains a leading cause of death and disability worldwide [1]. Timely restoration of blood flow (reperfusion) through strategies like thrombolysis and thrombectomy is crucial for salvaging ischemic brain tissue after stroke. Although the reperfusion process itself can trigger a complex pathological cascade, which leads to further brain damage, known as CIRI [1]. The principal mechanisms underlying CIRI encompass oxidative stress, inflammatory response, calcium overload, mitochondrial dysfunction, blood–brain barrier disruption, and programmed cell death such as apoptosis, necroptosis, autophagy, pyroptosis, etc. [1, 2].

Pyroptosis is a form of programmed cell death executed by gasdermins, a family of pore‐forming proteins [3]. Specific proteases cleave gasdermins, rendering them competent for pore formation [3]. The activation of the canonical gasdermin family member, gasdermin D (GSDMD), is linked to innate immune surveillance via the inflammasome [3]. In contrast, other gasdermins (GSDMA, GSDMB, GSDMC, and GSDME) are activated by inflammasome‐independent mechanisms [3]. Previous extensive studies indicate that CIRI promotes the assembly of the NLRP3 inflammasome. Activation of this inflammasome triggers caspase‐1, which subsequently cleaves the precursors of IL‐1ß and IL‐18 to their active forms [4]. Concurrently, caspase‐1 cleaves GSDMD, liberating its N‐terminal fragment (GSDMD‐NT) that forms pores in the cell membrane. These events collectively drive inflammatory responses and induce pyroptosis [5, 6, 7, 8, 9]. While recent studies have revealed that the GSDME pathway, activated by the apoptotic executioner caspase3, also contributes to pyroptosis following CIRI [10]. Nevertheless, the precise upstream and downstream regulatory mechanisms underlying this pathway remain poorly understood [10].

Leucine‐rich α‐2‐glycoprotein 1 (LRG1), a secreted member of the leucine‐rich repeat (LRR) family, is a glycoprotein [11]. Transcription of the LRG1 gene is primarily driven by the IL‐6/STAT3 signaling pathway [11]. Studies demonstrate that LRG1 deficiency attenuates the IL‐6/STAT3 cascade by reducing IL‐6 receptor (IL‐6R) expression in initial CD4‐positive lymphocytes [11]. In CIRI, LRG1 expression is upregulated and may contribute to regulating inflammatory responses and cell death processes [11], and LRG1 exacerbates ischemia/reperfusion injury by promoting apoptosis and autophagy, mediated through ALK1 upregulation via the TGFß‐Smad1/5 signaling pathway [12]. Conversely, LRG1 knockout confers significant neuroprotection against CIRI through multiple mechanisms: reducing blood–brain barrier (BBB) disruption by preserving cellular junctions, shifting microglial polarization from a pro‐inflammatory to a tissue‐reparative state, and decreasing neuronal and oligodendrocyte death, thereby attenuating post‐ischemic demyelination [13]. Furthermore, in acute myocardial infarction, hypoxia upregulates LRG1 and HIF‐1α expression, reducing H9c2 cardiomyocyte viability while promoting apoptosis and autophagy [14]. Collectively, these findings suggest that LRG1 may serve as a crucial regulator of neuroinflammation and pyroptosis in CIRI.

Influenced by concepts such as “preventing diseases before they occur” and “prevention is better than treatment”, primary and secondary stroke prevention have gained significant traction in recent years [15, 16]. Hyperbaric oxygen preconditioning (HBO‐PC), an emerging nonpharmacological approach, demonstrates potential value for both primary and secondary prevention of ischemic stroke. Its core mechanism involves activating endogenous protective pathways through intermittent hyperbaric oxygen exposure, enhancing tissue tolerance to subsequent ischemic–hypoxic injury [17]. Owing to fewer clinical side effects, HBO‐PC has been under intense investigation [18, 19].

The underlying protective mechanisms may involve HBO‐PC inhibiting the production of reactive oxygen species (ROS) [20], upregulating hypoxia‐inducible factor 1 (HIF‐1) [21], enhancing antioxidant enzyme activity [22], inhibiting the expression of pro‐inflammatory cytokines such as IL‐6 and IL‐1ß [22], reducing the activity of apoptotic protein caspase‐3 [22] and suppressing matrix metalloproteinase‐9 (MMP‐9) activity [23]. According to reports, in patients with acute ischemic stroke caused by large‐vessel occlusion in the anterior circulation who were candidates for endovascular treatment, normobaric hyperoxia yielded superior functional outcomes at 90 days compared with the sham normobaric hyperoxia, without raising safety concerns [24]. Nevertheless, the neuroprotective mechanisms of HBO‐PC, particularly the key signaling pathways involved, are not fully understood [25].

Based on the existing evidence, this study aims to investigate whether HBO‐PC alleviates CIRI by disrupting the LRG1‐mediated HIF‐1α/GSDME/IL‐6/STAT3 positive feedback loop, thereby inhibiting pyroptosis and promoting microglial polarization from a pro‐inflammatory to an anti‐inflammatory phenotype. Ultimately, we seek to provide a rationale for a novel stroke prevention and treatment strategy combining HBO‐PC with targeted molecular therapy, which may enhance the precision and reliability of HBO‐PC as a primary or secondary preventive approach in clinical settings.

## 2 Materials and Methods

### 2.1 Ethical Approval for Animal Experimental Protocol

All animal procedures were approved by the Ethics Committee of the 6th Medical Center, PLA General Hospital (No. JFJZYYDLYXZX, 2023–10) and reported in accordance with ARRIVE guidelines. Healthy adult male C57BL/6J mice were obtained from Spiff (Beijing) Biotechnology Co. Ltd. and housed under standard conditions (12 h light/dark cycle, controlled temperature, ad libitum food and water).

### 2.2 Animal Model of Cerebral Ischemia/Reperfusion Injury and HBO‐PC

Mice were randomly assigned to four groups: (1) Sham, (2) MCAO/R (middle cerebral artery occlusion/reperfusion), (3) HBO‐PC (hyperbaric oxygen preconditioning), (4) HBO‐PC + MCAO/R. The CIRI model was induced in 3‐month‐old mice using standardized transient MCAO/R [26]. Briefly, under 2% isoflurane anesthesia, the left common carotid, external carotid, and pterygopalatine arteries were ligated. A silicone‐coated monofilament was introduced via the external carotid stump and advanced to occlude the middle cerebral artery for 60 min, followed by withdrawal to allow reperfusion. Sham mice underwent identical surgery without occlusion. HBO‐PC consisted of exposure to 100% oxygen at 2.5 ATA for 1 h daily for 3 consecutive days prior to MCAO/R [27].

### 2.3 Drug Administration

For LRG1 knockdown studies, mice received intracerebroventricular injections of either siLRG1 or a scrambled control siRNA (GenePharma) prior to modeling/HBO‐PC, forming six groups [28]: (1) Scramble + Sham, (2) Scramble + MCAO/R, (3) siLRG1 + Sham, (4) siLRG1 + MCAO/R, (5) siLRG1 + HBO‐PC + Sham, (6) siLRG1 + HBO‐PC + MCAO/R.

### 2.4 Evaluation of Neurological Deficits

Neurological deficits were evaluated using the modified Neurological Severity Score (mNSS) [29], a composite score ranging from 0 to 18 (higher scores indicate greater impairment). The assessment covered motor, sensory, balance, and reflex functions. All tests were performed in triplicate under controlled, low‐light conditions to ensure consistency. Based on the total score, injury severity was categorized as mild (1–6), moderate (7–12), or severe (13–18).

### 2.5 Morris Water Maze

Spatial learning and memory were assessed using the Morris Water Maze [30]. Mice were trained over 4–7 days (four trials/day) in a pool (22°C ± 1°C) with a submerged platform. Escape latency and path length were recorded. A 60‐s probe trial (platform removed) was conducted 24 h after training to measure time/distance in the target quadrant and platform crossings. A visible‐platform test confirmed intact sensorimotor and visual function [31]. Environmental conditions were strictly controlled throughout.

### 2.6 Rotarod Test

Motor coordination and balance were evaluated using the rotarod test [32]. After a 3‐day adaptation period on a stationary or low‐speed (≤ 5 rpm) rod, mice underwent three trials per session at baseline (pre‐MCAO/R) and on days 1, 3, and 5 post‐MCAO/R. The latency to fall was recorded using either an accelerating protocol (starting at 4 rpm, increasing by 4 rpm/min) or a fixed‐speed mode (12–20 rpm). Data from mice that jumped voluntarily were excluded. Tests were conducted under controlled low‐noise conditions with protective padding in place [33, 34].

### 2.7 Adhesive Removal Test

Sensory and motor function were evaluated using the Neurobehavioral Adhesive Removal Test [35]. After a 3‐day acclimation period, a circular adhesive (5–7 mm) was applied to each forelimb. The time to perceive the adhesive and the time to completely remove it were recorded, with a maximum trial duration of 300 s. Each mouse underwent three trials per session (pre‐MCAO/R baseline and post‐operative days 1, 3, and 5) under controlled conditions. Identical adhesive batches were used, and testing was limited to twice weekly to minimize learning effects [36, 37].

### 2.8 Brain Infarct Volume Determination

After 24 h reperfusion, brains were sectioned and stained with 2% TTC. Infarct volumes were quantified with ImageJ software (v1.50g; NIH, USA) and calculated as: Relative Infarct Volume (%) = [(Contralateral Hemisphere Volume—Non‐infarcted Ipsilateral Tissue Volume)/(Contralateral Hemisphere Volume × 2)] × 100% [38].

### 2.9 Immunofluorescence Analysis

Brain sections (4–5 μm) from the hippocampal region were immunostained with primary antibodies against LRG1 (Proteintech, #13224‐1‐AP) at 1:200, GFAP (Abcam, #ab7260) at 1:200, NeuN (Abcam, #ab177487) at 1:200, Iba1 (Abcam, #ab178846) at 1:200, Olig2 (Abcam, #ab109186) at 1:100, IL‐6 (Abcam, #ab305225) at 1:100, IL‐10 (Abcam, #ab133575) at 1:100, Bax (Abcam, #ab216494) at 1:100, Bcl‐2 (Immunoway, #YM3041) at 1:100, and Caspase‐3 (Immunoway, #YM3431), followed by appropriate secondary antibodies and DAPI counterstain. Images were acquired by fluorescence/confocal microscopy and quantified with ImageJ [6].

### 2.10 Western Blot

After the mice were euthanized, 40 μg of brain tissue protein was collected from the striatum region on the ipsilateral side of the ischemia–reperfusion injury. Proteins were separated by SDS‐PAGE, transferred to membranes, and probed with antibodies against anti‐LRG1 (Proteintech, #1324‐1‐AP), anti‐IL‐6 (Abcam, #ab305225), anti‐STAT3 (Abcam, #ab68153), and anti‐p‐STAT3 (Abcam, #ab267373), Rabbit Polyclonal HIF‐1 alpha [Hydroxy Pro564] Antibody (cell signal, #3434t), Pro Caspase‐3 (5E1) Mouse mAb (ImmunoWay, #ym3431), Cleaved Caspase 3 Polyclonal antibody (Proteintech, #25128‐1‐ap), GSDME N‐terminal Rabbit pAb (ImmunoWay, #yt7990), GSDME Full length Rabbit pAb (abclonal, #a7432). Following TBS‐T washes, membranes were incubated with horseradish peroxidase‐conjugated IgG secondary antibody (1:5000) for 2 h at room temperature. β‐actin (Abcam, #ab8226) served as loading control. Bands were visualized by ECL and quantified with ImageJ [6].

### 2.11 Transmission Electron Microscopy (TEM)

Brain tissue was fixed in glutaraldehyde and osmium tetroxide, dehydrated, embedded, and sectioned. Ultrathin sections were stained and observed using a HITACHI H‐7500 TEM [6].

### 2.12 ELISA Analysis

Cytokine levels (IL‐6, TNF‐α, IL‐1ß, IL‐10) in the ischemic area were measured using commercial ELISA kits according to manufacturers' protocols [6].

### 2.13 Statistical Analysis

Data are presented as mean ± SEM and analyzed with GraphPad Prism 9.0. Normality and homogeneity of variance were tested. Two‐group comparisons used *t*‐test or Mann–Whitney *U* test. Multi‐group comparisons used one‐way ANOVA. *p* < 0.05 was considered significant.

## 3 Result

### 3.1 HBO‐PC Decreases Brain Damage and Alleviates Post‐Stroke Neurological Deficits

**FIGURE 1:**
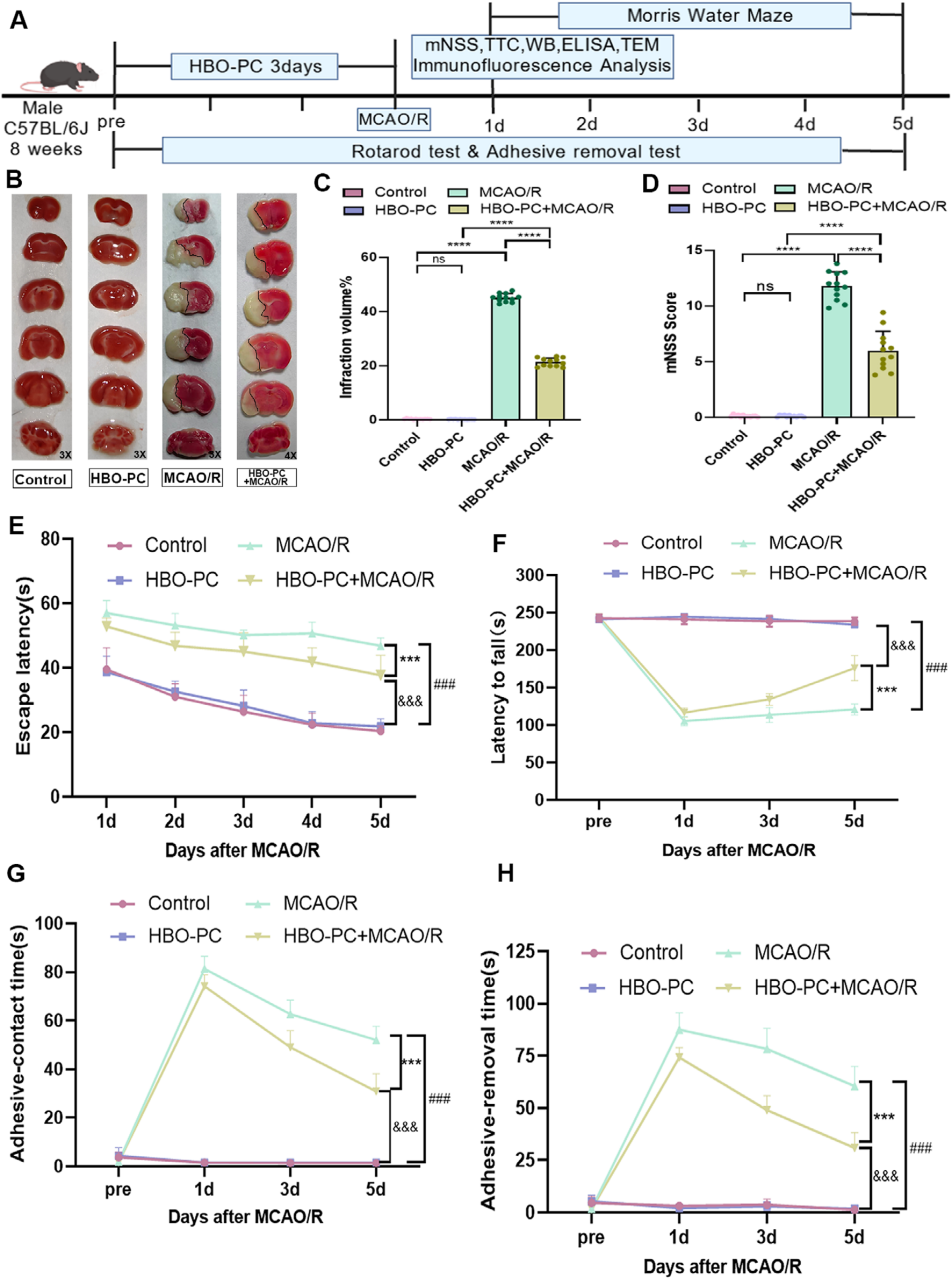
(A) The timeline of the first part of the experiment. (B) Triphenyl tetrazolium chloride (TTC) staining of brain sections at 24 h after sham operation or MCAO/R of the control group, HBO‐PC group, MCAO/R group, and HBO‐PC + MCAO/R group (*n* = 12 mice per group). (C) Volume statistics chart of cerebral infarction of brain sections at 24 h after sham operation or MCAO/R of the control group, HBO‐PC group, MCAO/R group and HBO‐PC + MCAO/R group (*n* = 12 mice per group). ns, there was no statistically significant difference between the two groups. ****p < 0.0001. (D) Modified neurological severity score (mNSS) test at 24 h after sham operation or MCAO/R of the control group, HBO‐PC group, MCAO/R group and HBO‐PC + MCAO/R group (*n* = 12 mice per group). ns, there was no statistically significant difference between the two groups. ****p < 0.0001. (E) The mean escape latency in the morris water maze at 1–5 days after sham operation or MCAO/R of the control group, HBO‐PC group, MCAO/R group and HBO‐PC + MCAO/R group (*n* = 12 mice per group). * represents a significant difference between the MCAO/R group and the HBO‐PC + MCAO/R group. # represents a significant difference between the HBO‐PC group and the HBO‐PC + MCAO/R group. & represents a significant difference between the control group and the MCAO/R group. ***, ###, and &&&*p* < 0.001. (F) Latency to fall in the rotarod test from pretraining day to Day 5 after sham operation or MCAO/R of the control group, HBO‐PC group, MCAO/R group and HBO‐PC + MCAO/R group (*n* = 12 mice per group). * represents a significant difference between the MCAO/R group and the HBO‐PC + MCAO/R group. # represents a significant difference between the HBO‐PC group and the HBO‐PC + MCAO/R group. & represents a significant difference between the control group and the MCAO/R group. ***, ### and &&&*p* < 0.001. (G) Time required to feel the adhesive tapes during adhesive contact test (contact time) from pretraining day to Day 5 after sham operation or MCAO/R of the control group, HBO‐PC group, MCAO/R group and HBO‐PC + MCAO/R group (*n* = 12 mice per group). * represents a significant difference between the MCAO/R group and the HBO‐PC + MCAO/R group. # represents a significant difference between the HBO‐PC group and the HBO‐PC + MCAO/R group. & represents a significant difference between the control group and the MCAO/R group. ***, ###, and &&&*p* < 0.001. (H) Time required to feel the adhesive tapes during adhesive removal test (removal time) from pretraining day to Day 5 after sham operation or MCAO/R of the control group, HBO‐PC group, MCAO/R group and HBO‐PC + MCAO/R group (*n* = 12 mice per group). * represents a significant difference between the MCAO/R group and the HBO‐PC + MCAO/R group. # represents a significant difference between the HBO‐PC group and the HBO‐PC + MCAO/R group. & represents a significant difference between the control group and the MCAO/R group. ***, ###, and &&&*p* < 0.001.

To examine the effects of HBO‐PC on CIRI, male C57BL/6J mice were subjected to HBO‐PC prior to MCAO/R. TTC staining revealed that HBO‐PC significantly reduced cerebral infarct volume compared with the MCAO/R group (Figure 1A–C). Neurological function, assessed by the mNSS, was markedly impaired after MCAO/R, and this deficit was significantly reversed by HBO‐PC (Figure 1D). In the Morris water maze, the MCAO/R group showed prolonged escape latencies during training days 1–5, whereas the HBO‐PC + MCAO/R group exhibited shorter latencies, indicating improved spatial learning and memory (Figure 1E). Motor coordination, tested by rotarod performance, was severely impaired in MCAO/R mice but significantly preserved in the HBO‐PC + MCAO/R group (Figure 1F). Similarly, in the adhesive‐removal test, both the time to perceive and the time to remove the adhesive were increased after MCAO/R, and these delays were substantially shortened by HBO‐PC (Figure 1G,H). Together, these results demonstrate that HBO‐PC alleviates cerebral infarction, neurological deficits, and motor‐cognitive impairments induced by CIRI.

### 3.2 HBO‐PC Suppresses LRG1 Expression in Multiple Neural Cell Types Following CIRI

**FIGURE 2:**
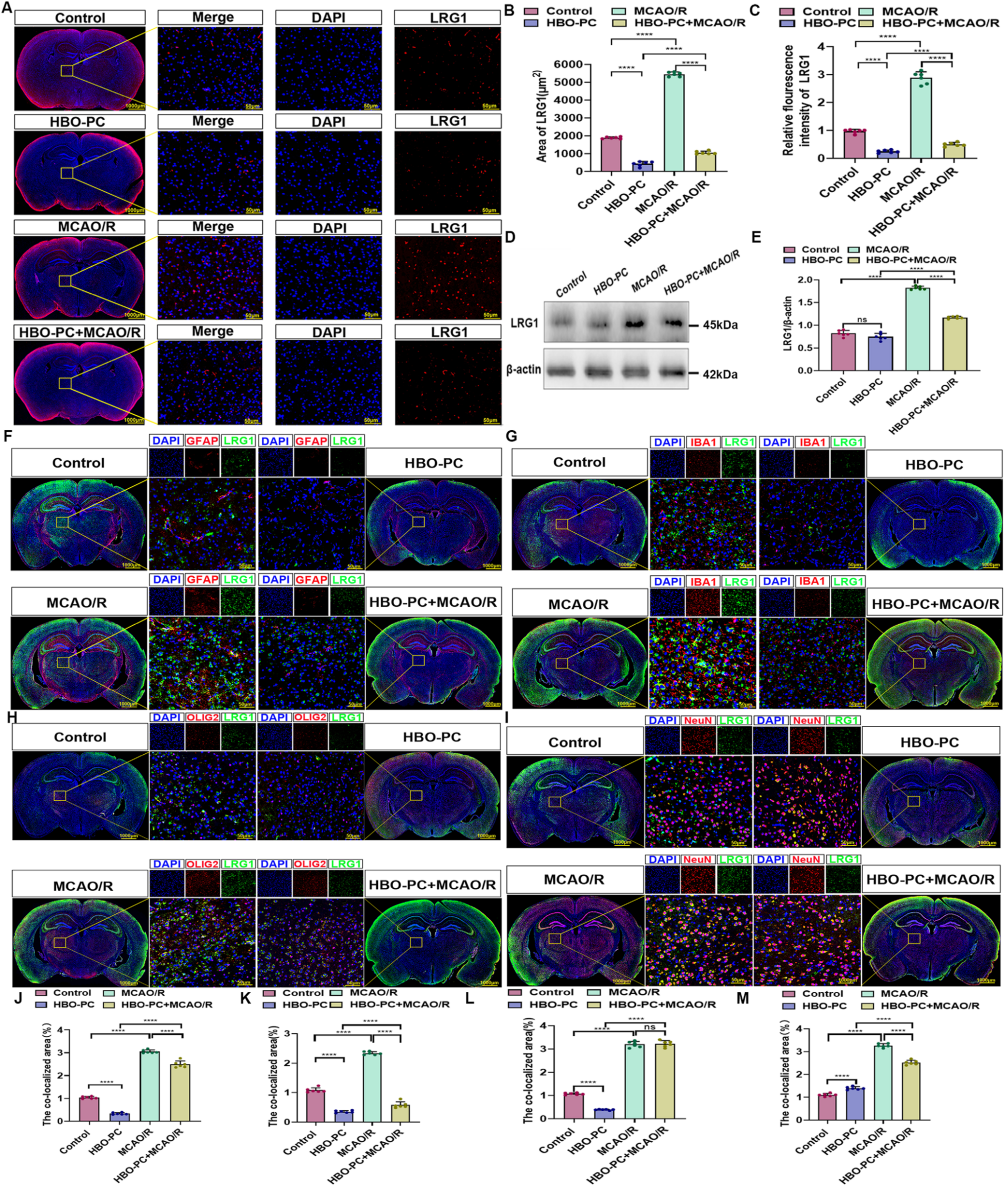
(A) Immunofluorescence staining was performed with LRG1 antibody (red) in brain sections. Nuclear fluorescent labeling with DAPI (blue). The results showed that the expression level of LRG1 increased in the MCAO/R group compared with the control group and decreased in the HBO + MCAO/R group compared with the MCAO/R group (*n* = 6 rats per group). Scale bars, 1000 μm and 50 μm. (B) The mean area of LRG1. The results showed that the expression level of LRG1 increased in the MCAO/R group compared with the control group and decreased in the HBO + MCAO/R group compared with the MCAO/R group (*n* = 6 rats per group). ****p < 0.0001; ns, there was no statistically significant difference between the two groups. (C) The relative fluorescence intensity of LRG1. The results showed that the expression level of LRG1 increased in the MCAO/R group compared with the control group and decreased in the HBO + MCAO/R group compared with the MCAO/R group (*n* = 6 rats per group). ****p < 0.0001; ns, there was no statistically significant difference between the two groups. (D) The western blotting of LRG1 in the area of cerebral ischemia–reperfusion injury. The results showed that the expression level of LRG1 increased in the MCAO/R group compared with the control group and decreased in the HBO + MCAO/R group compared with the MCAO/R group (*n* = 6 rats per group). (E) The relative expression of LRG1 (LRG1/ß‐actin). The results showed that the expression level of LRG1 increased in the MCAO/R group compared with the control group and decreased in the HBO + MCAO/R group compared with the MCAO/R group (*n* = 6 rats per group). ****p < 0.0001; ns, there was no statistically significant difference between the two groups. (F) Double immunofluorescence analysis was performed using antibodies against LRG1 (green) and GFAP (red) in brain sections. Nuclei were fluorescently labeled with DAPI (blue). Merged images show increased co‐localization of LRG1 and GFAP and increased co‐localization after cerebral ischemia–reperfusion injury and decreased co‐localization after Hyperbaric oxygen preconditioning (*n* = 6 rats per group). Scale bars, 1000 and 50 μm. (G) Double immunofluorescence analysis was performed using antibodies against LRG1 (green) and IBA1 (red) in brain sections. Nuclei were fluorescently labeled with DAPI (blue). Merged images show increased co‐localization of LRG1 and IBA1 and increased co‐localization after cerebral ischemia–reperfusion injury (*n* = 6 rats per group). Scale bars, 1000 and 50 μm. (H) Double immunofluorescence analysis was performed using antibodies against LRG1 (green) and OLIG2 (red) in brain sections. Nuclei were fluorescently labeled with DAPI (blue). Merged images show increased co‐localization of LRG1 and OLIG2 and increased co‐localization after cerebral ischemia–reperfusion injury and decreased co‐localization after Hyperbaric oxygen preconditioning (*n* = 6 rats per group). Scale bars, 1000 and 50 μm. (I) Double immunofluorescence analysis was performed using antibodies against LRG1 (green) and NeuN (red) in brain sections. Nuclei were fluorescently labeled with DAPI (blue). Merged images show increased co‐localization of LRG1 and NeuN, increased co‐localization after cerebral ischemia–reperfusion injury and decreased co‐localization after Hyperbaric oxygen preconditioning (*n* = 6 rats per group). Scale bars, 1000 and 50 μm. (J) Bar chart showing the total area of co‐localized expression of LRG1 and GFAP (*n* = 6 in each group). ****p < 0.0001. (K) Bar chart showing the total area of co‐localized expression of LRG1 and IBA1 (*n* = 6 in each group). ****p < 0.0001; ns, there was no statistically significant difference between the two groups. (L) Bar chart showing the total area of co‐localized expression of LRG1 and OLIG2 (*n* = 6 in each group). ****p < 0.0001. (M) Bar chart showing the total area of co‐localized expression of LRG1 and NeuN (*n* = 6 in each group). ****p < 0.0001.

Based on previous findings of elevated LRG1 after cerebral ischemia–reperfusion injury [26], we examined the effect of HBO‐PC on LRG1 expression. Immunofluorescence and Western blot analyses both showed that LRG1 was significantly upregulated in the MCAO/R group compared to controls, and that HBO‐PC treatment markedly suppressed this increase (Figure 2A–E). We also performed immunofluorescence to qualitatively and quantitatively analyze the relative fluorescence intensity of LRG1 in the cortical region across the four groups. The results showed no significant difference in LRG1 expression in the cortex among the four groups (Figure S3T–U).

To determine LRG1 expression in specific neural cells, dual‐label immunofluorescence was performed. CIRI increased LRG1 in astrocytes, microglia, neurons, and oligodendrocytes (Figure 2F–M). HBO‐PC significantly reduced LRG1 expression in neurons, microglia, and astrocytes, with the strongest inhibitory effect observed in neurons and microglia (Figure 2F,G,I–K,M).

### 3.3 HBO‐PC Suppresses Neuroinflammation

**FIGURE 3:**
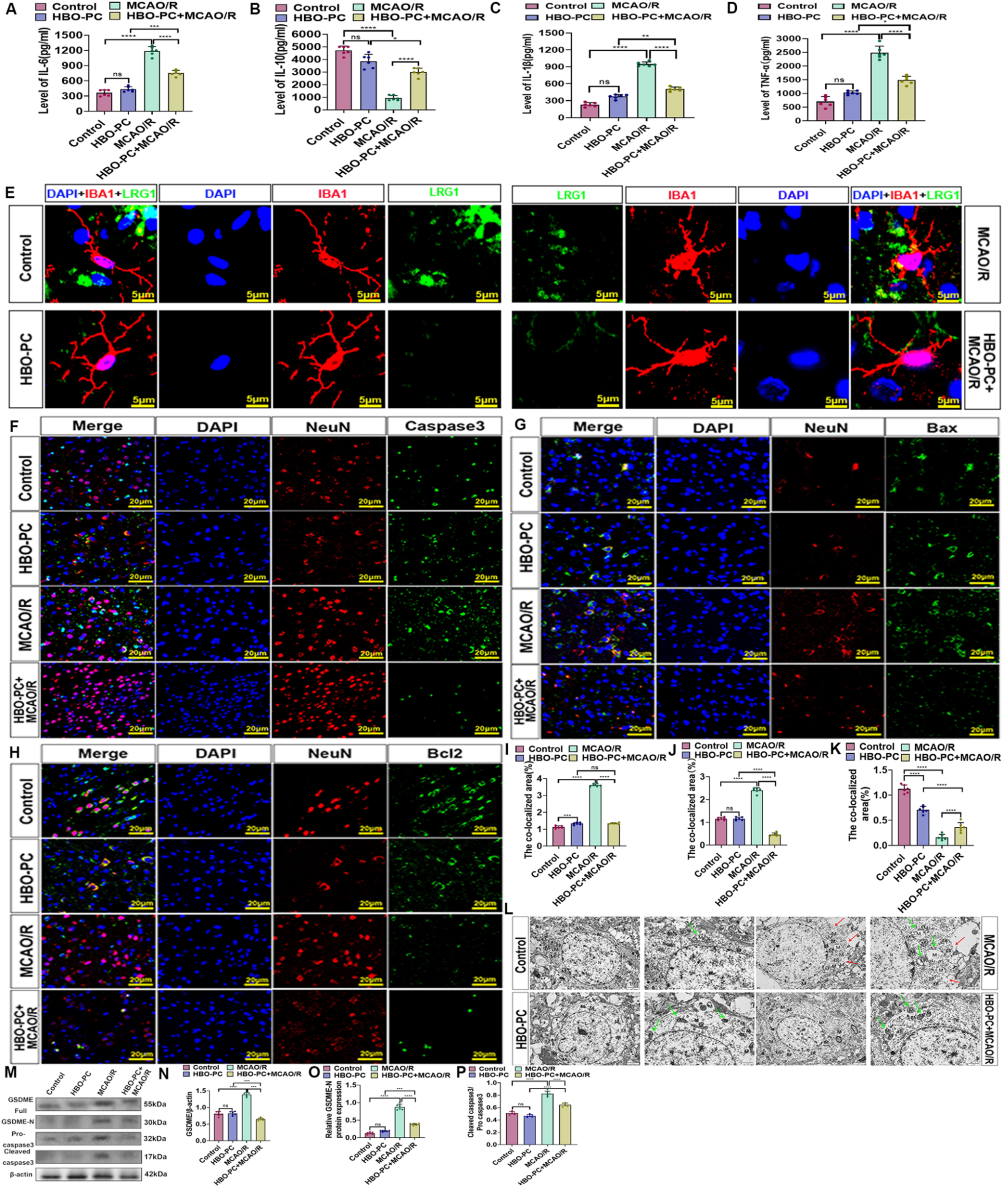
(A) ELISA of IL‐6. The bar chart of IL‐6 concentration levels, ****p < 0.0001; ***p < 0.001; ns, there was no statistically significant difference between the two groups. (B) ELISA of IL‐10. The bar chart of IL‐6 concentration levels, ****p < 0.0001; *p < 0.05; ns, there was no statistically significant difference between the two groups. (C) ELISA of IL‐1β. The bar chart of IL‐6 concentration levels, ****p < 0.0001; **p < 0.01; ns, there was no statistically significant difference between the two groups. (D) ELISA of TNF‐α. The bar chart of IL‐6 concentration levels, ****p < 0.0001; *p < 0.05; ns, there was no statistically significant difference between the two groups. (E) Double immunofluorescence analysis was performed using antibodies against IBA1 (red) and LRG1 (green) in brain sections. Nuclei were fluorescently labeled with DAPI (blue). Scale bars, 5 μm. (F) Double immunofluorescence analysis was performed using antibodies against Caspase3 (green) and NEUN (red) in brain sections. Nuclei were fluorescently labeled with DAPI (blue). Merged images show increased co‐localization of Caspase3 and NEUN increased co‐localization after cerebral ischemia–reperfusion injury (*n* = 6 rats per group). Scale bars, 20 μm. (G) Double immunofluorescence analysis was performed using antibodies against Bax (green) and NEUN (red) in brain sections. Nuclei were fluorescently labeled with DAPI (blue). Merged images show increased co‐localization of Bax and NEUN increased co‐localization after cerebral ischemia–reperfusion injury (*n* = 6 rats per group). Scale bars, 20 μm. (H) Double immunofluorescence analysis was performed using antibodies against Bcl‐2 (green) and NEUN (red) in brain sections. Nuclei were fluorescently labeled with DAPI (blue). Merged images show increased co‐localization of Bcl‐2 and NEUN increased co‐localization after cerebral ischemia–reperfusion injury (*n* = 6 rats per group). Scale bars, 20 μm. (I) Bar chart showing the total area of co‐localized expression of Caspase3 and NEUN (*n* = 6 in each group). ****p < 0.0001; ns, there was no statistically significant difference between the two groups. (J) Bar chart showing the total area of co‐localized expression of Bax and NEUN (*n* = 6 in each group). ****p < 0.0001. (K) Bar chart showing the total area of co‐localized expression of Bcl‐2 and NEUN (*n* = 6 in each group). ****p < 0.0001; ns, there was no statistically significant difference between the two groups. (L) Electron microscope image of mitochondrial apoptosis. N, nucleus; M, mitochondria in the cytoplasm; ER, endoplasmic reticulum; Lp, occasionally seen lipid droplet bodies in the cytoplasm, red represents the GSDME pores of cell pyroptosis. Scale bars: 2 μm and 500 nm. The red arrow points to the cell pyroptosis pore, and the green arrow points to the mitochondrion. (M) The western blotting of GSDME full length, GSDME‐N, Pro caspsae3 and cleaved caspase3 in the area of cerebral ischemia–reperfusion injury. The results showed that the expression level of GSDME full length, GSDME‐N, Pro caspsae3 and cleaved caspase3 increased in the MCAO/R group compared with the control group and decreased in the HBO + MCAO/R group compared with the MCAO/R group (*n* = 6 rats per group). (N) The GSDME/ß‐actin (fold). The results showed that the expression level of GSDME full length increased in MCAO/R group compared with the control group and decreased in HBO + MCAO/R group compared with the MCAO/R group (*n* = 6 rats per group). ***p < 0.001; ****p < 0.0001; ns, there was no statistically significant difference between the two groups. (O) The GSDME‐N/ß‐actin (fold). The results showed that the expression level of GSDME‐N increased in MCAO/R group compared with the control group and decreased in HBO + MCAO/R group compared with the MCAO/R group (*n* = 6 rats per group). ***p < 0.001; ****p < 0.0001; ns, there was no statistically significant difference between the two groups. (P) The Cleaved caspase3/Pro caspase3 (fold). The results showed that the expression level of Cleaved caspase3/Pro caspase3 increased in the MCAO/R group compared with the control group and decreased in the HBO + MCAO/R group compared with the MCAO/R group (*n* = 6 rats per group). ns, there was no statistically significant difference between the two groups; ****p < 0.0001.

We next assessed how HBO‐PC influences neural cell function after CIRI. ELISA showed that CIRI increased pro‐inflammatory cytokines (IL‐6, IL‐1β, TNF‐α) and decreased the anti‐inflammatory IL‐10, changes that were reversed by HBO‐PC (Figure 3A–D). Microglial morphology shifted from a ramified, anti‐inflammatory (M2) phenotype in controls and HBO‐PC groups to an amoeboid, pro‐inflammatory (M1) state after MCAO/R; HBO‐PC restored the M2‐like morphology (Figure 3E). Immunofluorescence further revealed that CIRI elevated IL‐6 and reduced IL‐10 in both neurons and microglia, effects that were significantly attenuated by HBO‐PC (Figure S1A–H). These results indicate that HBO‐PC effectively suppresses neuroinflammation in key neural cell types.

### 3.4 HBO‐PC Suppresses the Transition From Mitochondrial Apoptosis to GSDME‐Mediated Pyroptosis Induced by CIRI

To elucidate the mechanisms by which HBO‐PC affects neuronal fate after CIRI, we examined proteins associated with apoptosis and pyroptosis. Immunofluorescence showed that CIRI increased neuronal expression of the pro‐apoptotic proteins Bax and caspase‐3, while decreasing the anti‐apoptotic protein Bcl‐2. HBO‐PC significantly reversed these changes (Figure 3F–K). Ultrastructural analysis by electron microscopy revealed that MCAO/R induced mitochondrial swelling, cristae disruption, and neuronal pyroptosis (cell swelling, membrane pores). These pathological alterations were markedly ameliorated by HBO‐PC (Figure 3L).

Given that GSDME acts as a molecular switch linking apoptosis to pyroptosis [39], we assessed its expression. Western blot analysis demonstrated that MCAO/R upregulated full‐length GSDME, its N‐terminal fragment (GSDME‐N), and both cleaved and Pro caspase‐3. HBO‐PC significantly attenuated these increases (Figure 3M–P). These findings indicate that HBO‐PC inhibits the CIRI‐induced shift from mitochondrial apoptosis to GSDME‐mediated pyroptosis.

### 3.5 HBO‐PC Confers Neuroprotection to Alleviate CIRI Potentially Through the Inhibition of LRG1

**FIGURE 4:**
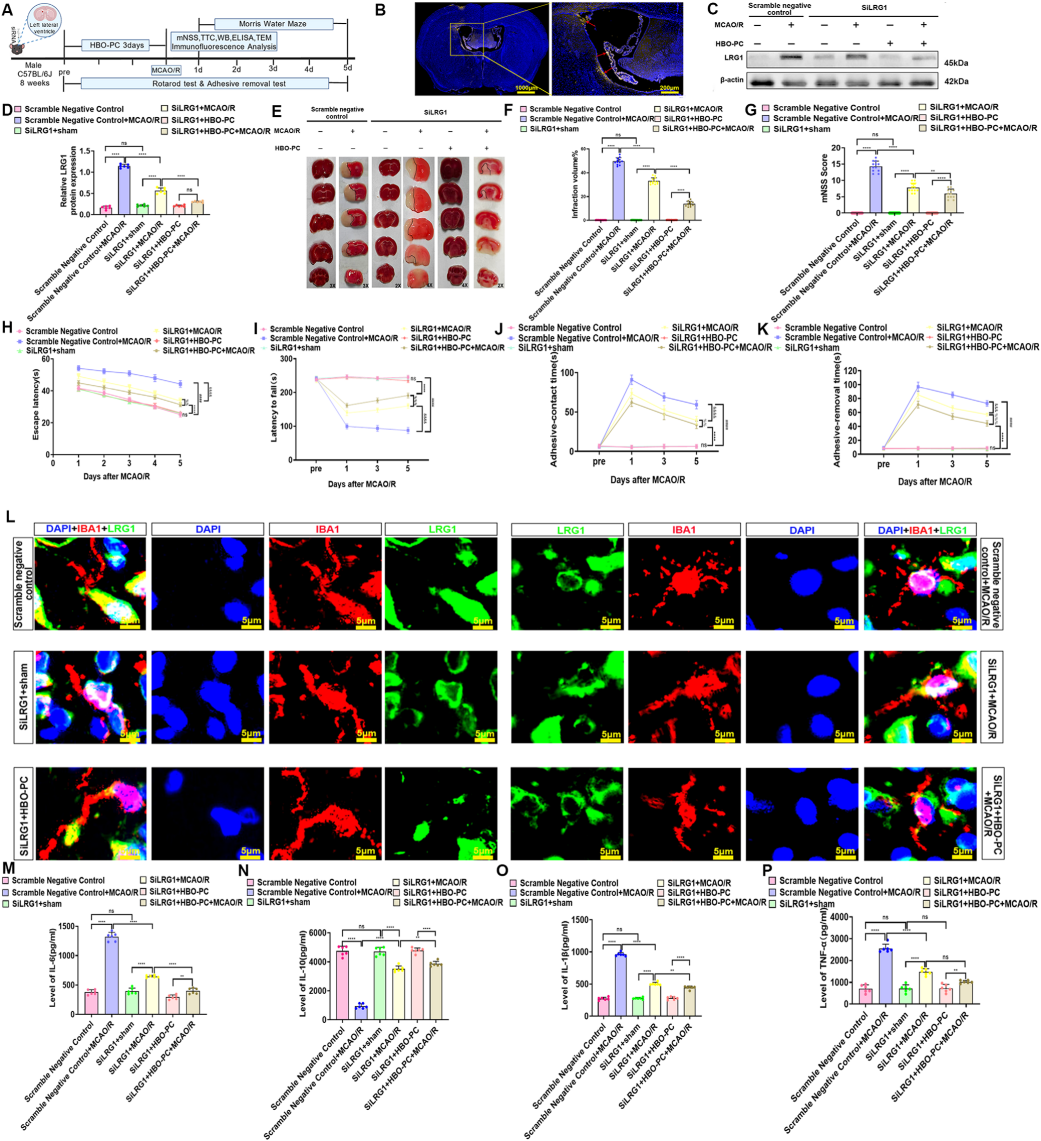
(A) The timeline of the sirna injection part experiment. (B) This immunofluorescence image depicts the distribution after siRNA injection. The nucleus is stained blue, and the delivered siRNA is shown in yellow. The successful injection into the left ventricle is indicated by the red arrows. Scale bars: 1000 μm and 200 μm. (C) The western blotting of LRG1 in the area of cerebral ischemia–reperfusion injury (*n* = 6 rats per group). (D) The statistical chart of LRG1/ß‐actin (fold). ****p < 0.0001; ns, there was no statistically significant difference between the two groups. (E) Triphenyl tetrazolium chloride (TTC) staining of brain sections (*n* = 12 mice per group). (F) Volume statistics chart of cerebral infarction of brain sections (*n* = 12 mice per group). ns, there was no statistically significant difference between the two groups. ****p < 0.0001. (G) Modified neurological severity score (mNSS) test (*n* = 12 mice per group). ns, there was no statistically significant difference between the two groups. ****p < 0.0001; **p < 0.01; ns, there was no statistically significant difference between the two groups. (H) The mean escape latency in the morris water maze (*n* = 12 mice per group). * represents a significant difference between the siLRG1 + MCAO/R group and the siLRG1 + HBO‐PC + MCAO/R group. # represents a significant difference between the scramble negative control group and the scramble negative control+ MCAO/R group. & represents a significant difference between the scramble negative control+ MCAO/R and the siLRG1+ MCAO/R group. % represents a significant difference between the siLRG1+ MCAO/R and the siLRG1 + HBO‐PC+ MCAO/R group. ****, #### and &&&&*p* < 0.0001; %%*p* < 0.01. ns, there was no statistically significant difference between the two groups; ns, there was no statistically significant difference between the two groups. (I) Latency to fall in the rotarod test (*n* = 12 mice per group). * represents a significant difference between the siLRG1 + MCAO/R group and the siLRG1 + HBO‐PC + MCAO/R group. # represents a significant difference between the scramble negative control group and the scramble negative control+ MCAO/R group. & represents a significant difference between the scramble negative control+ MCAO/R and the siLRG1+ MCAO/R group. % represents a significant difference between the siLRG1+ MCAO/R and the siLRG1 + HBO‐PC+ MCAO/R group. ****, #### and &&&&*p* < 0.0001; %%%*p* < 0.001. ns, there was no statistically significant difference between the two groups. (J) Time required to feel the adhesive tapes during adhesive contact test (contact time) (*n* = 12 mice per group). * represents a significant difference between the siLRG1 + MCAO/R group and the siLRG1 + HBO‐PC + MCAO/R group. # represents a significant difference between the scramble negative control group and the scramble negative control + MCAO/R group. & represents a significant difference between the scramble negative control + MCAO/R and the siLRG1 + MCAO/R group. % represents a significant difference between the siLRG1 + MCAO/R and the siLRG1 + HBO‐PC + MCAO/R group. ****, #### and &&&&*p* < 0.0001; %%*p* < 0.01; ns, there was no statistically significant difference between the two groups. (K) Time required to feel the adhesive tapes during adhesive removal test (removal time) (*n* = 12 mice per group). * represents a significant difference between the siLRG1 + MCAO/R group and the siLRG1 + HBO‐PC + MCAO/R group. # represents a significant difference between the scramble negative control group and the scramble negative control + MCAO/R group. & represents a significant difference between the scramble negative control + MCAO/R and the siLRG1 + MCAO/R group. % represents a significant difference between the siLRG1 + MCAO/R and the siLRG1 + HBO‐PC + MCAO/R group. ****, #### and &&&&*p* < 0.0001; %%*p* < 0.01; ns, there was no statistically significant difference between the two groups. (L) Double immunofluorescence analysis was performed using antibodies against IBA1 (red) and LRG1 (green) in brain sections. Nuclei were fluorescently labeled with DAPI (blue). Scale bars, 5 μm. (M) ELISA of IL‐6. The bar chart of IL‐6 concentration levels, ****p < 0.0001; **p < 0.01; ns, there was no statistically significant difference between the two groups. (N) ELISA of IL‐10. The bar chart of IL‐6 concentration levels, ****p < 0.0001; **p < 0.01; ns, there was no statistically significant difference between the two groups. (O) ELISA of IL‐1β. The bar chart of IL‐6 concentration levels, ****p < 0.0001; **p < 0.01; ns, there was no statistically significant difference between the two groups. (P) ELISA of TNF‐α. The bar chart of IL‐6 concentration levels, ****p < 0.0001; **p < 0.01; ns, there was no statistically significant difference between the two groups.

To determine whether the neuroprotective effects of HBO‐PC may be associated with the inhibition of LRG1, we performed stereotaxic injection of siLRG1 into the left cerebral ventricle of mice prior to group modeling and analysis (Figure 4A). The efficacy of siRNA delivery was initially confirmed by immunofluorescence, which showed successful ventricular injection (Figure 4B, Figure S2A,B). Subsequent Western blot and immunofluorescence quantification revealed that LRG1 was significantly knocked down in the siLRG1 + MCAO/R group relative to the scramble control. Notably, the addition of HBO‐PC (siLRG1 + HBO‐PC + MCAO/R) resulted in a further decrease in LRG1 levels, indicating an additive effect (Figure 4C,D, Figure S2C,D).

To comprehensively evaluate neurological recovery and pathological outcomes, we employed a battery of tests including TTC staining, the modified neurological severity score (mNSS), and behavioral assays. TTC staining revealed a parallel stepwise reduction in cerebral infarction volume across the treatment groups (Figure 4E,F). Consistently across all assessments, both LRG1 knockdown and HBO‐PC conferred significant benefits, with the combined intervention (siLRG1 + HBO‐PC + MCAO/R) yielding the most pronounced effects. Specifically, the siLRG1 + MCAO/R group showed significant alleviation compared to the Scramble + MCAO/R control, as evidenced by reduced mNSS scores, shorter escape latency in the Morris water maze, longer fall latency on the rotarod, and decreased adhesive removal time (Figure 4G,H–K). These alleviations were further enhanced in the siLRG1 + HBO‐PC + MCAO/R group. Collectively, these results support that the neuroprotective effects of HBO‐PC against CIRI are potentially linked to the downregulation of LRG1 expression and the inhibition of LRG1 may contribute to the beneficial actions of HBO‐PC in this pathological context.

### 3.6 HBO‐PC Mitigates Neuroinflammation and Suppresses the Transition From Mitochondrial Apoptosis to GSDME‐Mediated Pyroptosis by Inhibiting LRG1

To determine whether HBO‐PC alleviates neuroinflammation via LRG1 suppression, we assessed microglial morphology and cytokine levels. Immunofluorescence showed that microglia in the Scramble+MCAO/R group exhibited an amoeboid, M1 phenotype, whereas LRG1 knockdown (siLRG1 + MCAO/R) and combined treatment (siLRG1 + HBO‐PC + MCAO/R) promoted an elongated, M2‐like morphology (Figure 4L). ELISA confirmed that MCAO/R increased pro‐inflammatory cytokines (IL‐6, IL‐1β, TNF‐α) and decreased IL‐10. These changes were partially reversed by siLRG1 and further normalized by the addition of HBO‐PC (Figure 4M–P).

**FIGURE 5:**
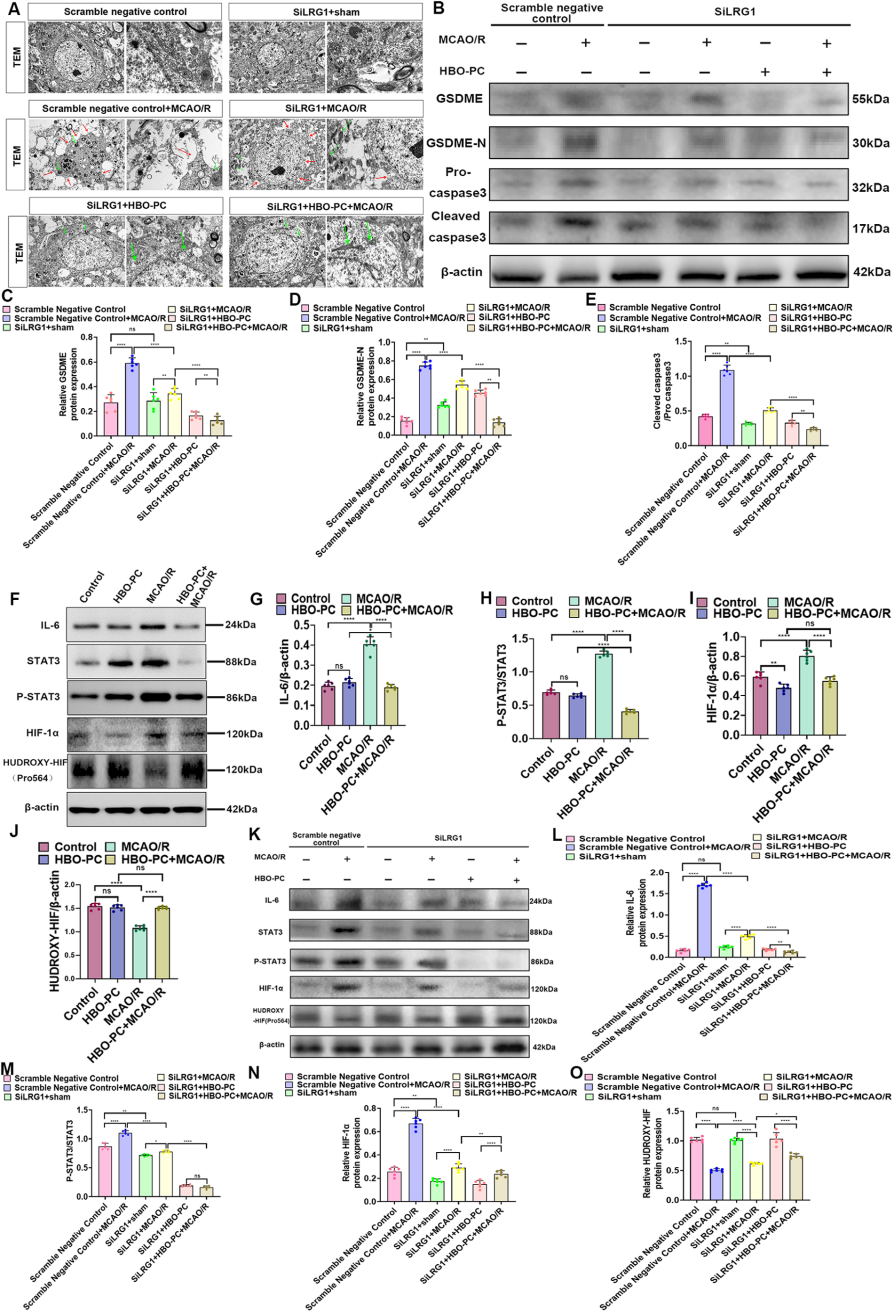
(A) Electron microscope image of cell pyroptosis. ER, endoplasmic reticulum; M, mitochondria in the cytoplasm; N, nucleus; Lp, occasionally seen lipid droplet bodies in the cytoplasm, red represents the GSDME pores of cell pyroptosis. Scale bars: 2 μm and 500 nm. The red arrow points to the cell pyroptosis pore, and the green arrow points to the mitochondrion. (B) The western blotting of GSDME full length, GSDME‐N, procaspase‐3, and cleaved caspase‐3 in area of cerebral ischemia–reperfusion injury (*n* = 6 rats per group). (C) The GSDME/ß‐actin (fold) (*n* = 6 rats per group). **p < 0.01; ****p < 0.0001; ns, there was no statistically significant difference between the two groups. (D) The GSDME‐N/ß‐actin (fold) (*n* = 6 rats per group). **p < 0.01; ****p < 0.0001; ns, there was no statistically significant difference between the two groups. (E) The Cleaved caspase3/Pro caspase3 (fold) (*n* = 6 rats per group). **p < 0.01; ****p < 0.0001. (F) The western blotting of IL‐6, STAT3, P‐STAT3, HIF‐1α, and HUDROXY‐HIF(Pro564) in the area of cerebral ischemia–reperfusion injury (*n* = 6 rats per group). (G) The IL‐6/ß‐actin (fold) (*n* = 6 rats per group). ****p < 0.0001; ns, there was no statistically significant difference between the two groups. (H) The P‐STAT3/STAT3 (fold) (*n* = 6 rats per group). ****p < 0.0001; ns, there was no statistically significant difference between the two groups. (I) The HIF‐1α/ß‐actin (fold) (*n* = 6 rats per group). **p < 0.01; ****p < 0.0001; ns, there was no statistically significant difference between the two groups. (J) The HUDROXY‐HIF /ß‐actin (fold) (*n* = 6 rats per group). ****p < 0.0001; ns, there was no statistically significant difference between the two groups. (K) The western blotting of IL‐6, STAT3, P‐STAT3, HIF‐1α, and HUDROXY‐HIF(Pro564) in the area of cerebral ischemia–reperfusion injury (*n* = 6 rats per group). (L) The IL‐6/ß‐actin (fold) (*n* = 6 rats per group). **p < 0.01 ****p < 0.0001; ns, there was no statistically significant difference between the two groups. (M) The P‐STAT3/STAT3 (fold) (*n* = 6 rats per group). *p < 0.05; **p < 0.01; ****p < 0.0001. (N) The HIF‐1α/ß‐actin (fold) (*n* = 6 rats per group). **p < 0.01; ****p < 0.0001. (O) The HUDROXY‐HIF /ß‐actin (fold) (*n* = 6 rats per group). *p < 0.05; ****p < 0.0001; ns, there was no statistically significant difference between the two groups.

Electron microscopy revealed graded ultrastructural protection. The Scramble+MCAO/R group showed mitochondrial swelling, cristae loss, and neuronal pyroptosis (nuclear condensation, organelle vacuolation, membrane pores). These features were moderately improved in the siLRG1 + MCAO/R group and nearly normalized in the siLRG1 + HBO‐PC + MCAO/R group (Figure 5A).

Western blot analysis indicated that LRG1 knockdown significantly reduced levels of full‐length GSDME, GSDME‐N, cleaved caspase‐3, and Pro caspase‐3 compared to Scramble+MCAO/R, with the greatest reduction in the siLRG1 + HBO‐PC + MCAO/R group (Figure 5B–E). These data demonstrate that HBO‐PC attenuates neuroinflammation and suppresses the shift to GSDME‐mediated pyroptosis by inhibiting LRG1.

### 3.7 HBO‐PC Disrupts the LRG1‐Mediated HIF1α‐GSDME‐IL‐6‐STAT3 Positive Feedback Loop to Attenuate Neuroinflammation and Neuron Pyroptosis

Existing literature shows that HBO‐PC reduces HIF‐1α levels in ischemic brain tissue [40] whereas LRG1 upregulation inhibits HIF‐1α hydroxylation, resulting in its stabilization and subsequent activation of pyroptosis and inflammatory mediators including IL‐6 [14, 41]. Since LRG1 transcription is primarily activated by the IL‐6/STAT3 pathway [11], and our data demonstrate substantial IL‐6 elevation in neurons after injury—an effect reversed by HBO‐PC (Figure 3F–M)—we propose that CIRI triggers an LRG1‐driven HIF‐1α–GSDME–IL‐6–STAT3 positive feedback circuit. This cycle sustains inflammatory signaling and neural injury, and HBO‐PC exerts its neuroprotective role primarily by disrupting this self‐amplifying loop. To validate this hypothesis, we assessed the expression of key signaling proteins by Western Blot. It was indicated that compared with the control group, the MCAO/R group exhibited a significant increase in HIF‐1α expression along with a marked decrease in its hydroxylation level. In addition, the MCAO/R group showed significantly elevated expression of IL‐6, STAT3, and phosphorylated STAT3 (P‐STAT3) compared to the control group. These changes were effectively reversed by HBO‐PC (HBO‐PC + MCAO/R group) (Figure 5F–J). Moreover, LRG1 knockdown (siLRG1 + MCAO/R group) led to a significant decrease in IL‐6, STAT3, P‐STAT3, and HIF‐1α compared to the scramble control, with the greatest suppression observed in the siLRG1 + HBO‐PC + MCAO/R group. This indicates that HBO‐PC disrupts the IL‐6–STAT3–LRG1 positive feedback loop, thereby attenuating pyroptosis (Figure 5K–O). Furthermore, we compared the siLRG1 + MCAO/R group with the HBO‐PC + MCAO/R group using TTC staining, mNSS, behavioral tests as well as assessments of LRG1 expression, neuroinflammation‐related proteins and pyroptosis‐related proteins. The results showed there were no statistically significant differences between the group with inhibition of LRG1 expression before CIRI (siLRG1 + MCAO/R) and the group with HBO‐PC before CIRI (HBO‐PC + MCAO/R) across all measurements. This further suggests that HBO‐PC may mainly alleviate CIRI by inhibiting the LRG1 pathway (Figure S3A–S). Integrating these results with previous cellular pyroptosis data, we conclude that HBO‐PC inhibits pyroptosis and ameliorates CIRI by interrupting the LRG1‐mediated HIF‐1α–GSDME–IL‐6–STAT3 positive feedback cycle.

## 4 Discussion

CIRI is a complex pathophysiological process involving multiple mechanisms, including oxidative stress, inflammatory responses, calcium overload, mitochondrial dysfunction, blood–brain barrier disruption, and programmed cell death [1]. Pyroptosis, a recently identified form of programmed cell death characterized by its inflammatory nature, has been shown to play a critical role in the initiation and progression of inflammatory injury [41]. Growing evidence suggests that cellular pyroptosis and the accompanying inflammatory response significantly contribute to the exacerbation of ischemic brain damage. Therefore, inhibition of pyroptosis may represent a promising therapeutic strategy to mitigate injury following cerebral ischemia [42].

HBO‐PC is an emerging nonpharmacological prophylactic strategy with potential for both primary and secondary prevention of ischemic stroke [17]. Its core mechanism relies on inducing endogenous protective pathways via intermittent hyperbaric oxygen exposure, thereby increasing tissue resilience to subsequent ischemic–hypoxic insults [17]. Research has demonstrated that HBO‐PC significantly mitigates brain injury in murine models of CIRI, as evidenced by reduced cerebral infarct volume, attenuated brain edema, and decreased neuronal apoptosis, collectively leading to alleviated neurological outcomes [27]. The underlying protective mechanisms are multifaceted and may include: suppression of reactive oxygen species (ROS) production [20], upregulation of hypoxia‐inducible factor 1 (HIF‐1) [21], enhancement of antioxidant enzyme activity [22], inhibition of pro‐inflammatory cytokines such as IL‐6 and IL‐1ß [22], reduction in caspase‐3‐mediated apoptotic activity [22], and decreased matrix metalloproteinase‐9 (MMP‐9) activity [43]. Furthermore, a regimen of five consecutive days of HBO‐PC (2.5 ATA, 100% O₂, 1 h/day) has been shown to downregulate HIF‐1α expression in ischemic brain tissue, thereby preserving blood–brain barrier integrity following ischemia/reperfusion injury [23]. Although the protective effects of HBO‐PC against CIRI have been extensively documented, a central pathway that integrates these diverse changes remains elusive.

**FIGURE 6:**
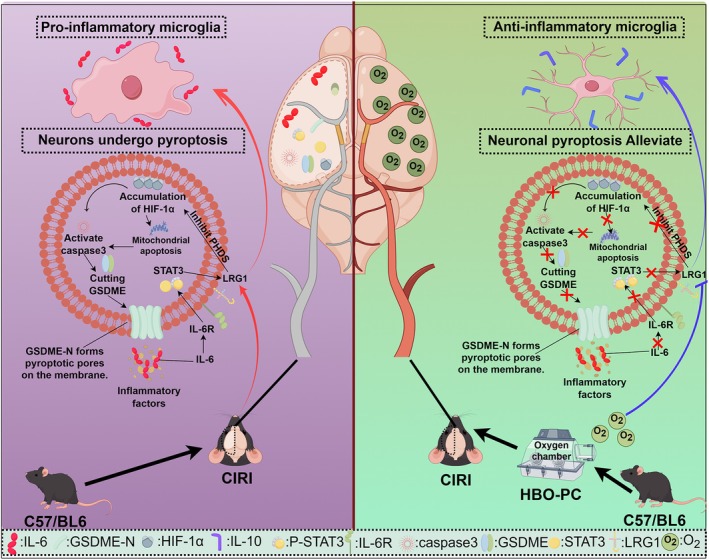
Hyperbaric oxygen preconditioning alleviates neuroinflammation and pyroptosis after cerebral ischemia–reperfusion by targeting the LRG1/HIF1α/GSDME axis.

Our study suggests that HBO‐PC may reprogram neuroimmune metabolism by interfering with the LRG1‐HIF‐1α‐IL‐6‐STAT3 amplification loop thereby reducing pyroptosis and CIRI. Based on existing literature and the observations of this study, LRG1 appears to be a key target through which HBO‐PC improves neurological recovery after CIRI. After injury, LRG1 is thought to inhibit prolyl hydroxylase activity thereby blocking the hydroxylation of HIF‐1α, reducing its degradation through the ubiquitination pathway and promoting the accumulation of HIF‐1α, a process that may further activate caspase‐3, leading to GSDME protein cleavage, inducing pyroptosis and triggering the release of the pro‐inflammatory cytokine IL‐6. The released IL‐6 may activate STAT3, which in turn may upregulate LRG1 expression, forming a putative self‐amplifying vicious cycle that exacerbates CIRI. The neuroprotective effect of HBO‐PC may partly stem from the inhibition of LRG1 expression, thereby potentially breaking this detrimental feedback loop. Our findings also imply that the inhibition of LRG1 by HBO‐PC may induce a phenotypic shift of microglia from a pro‐inflammatory to an anti‐inflammatory phenotype, a change that may serve as an additional mechanism to synergistically alleviate severe CIRI (Figure 6). These observations provide a potential innovative strategy for the prevention and treatment of stroke, namely combining HBO‐PC with targeted molecular intervention, a strategy that may help improve the reliability and precision of HBO‐PC as a clinical approach for primary or secondary stroke prevention.

LRG1 is a secreted glycoprotein of the leucine‐rich repeat (LRR) family. Its transcription is primarily driven by the IL‐6/STAT3 signaling pathway, with which it forms a positive feedback cascade [11]. However, it remains unknown whether this mechanism operates in the context of CIRI. Previous studies have established that LRG1 upregulation is associated with increased cerebral infarction volume, neuronal apoptosis, and enhanced autophagy, thereby exacerbating CIRI [44, 45]. Notably, LRG1 levels in ischemic brain tissue continue to rise with prolonged reperfusion time [26]. Its upregulation across diverse cell types indicates a multifaceted role in disease progression [26]. Inhibiting LRG1 expression ameliorates CIRI by strengthening blood–brain barrier integrity, modulating microglia and macrophage states, and reducing neuronal and oligodendrocyte death [26]. Besides, previous research on LRG1 in CIRI has predominantly focused on the classical pathway, wherein LRG1 upregulates ALK1 via the transforming growth factor ß‐Smad1/5 signaling pathway to promote apoptosis and autophagy, thereby exacerbating injury [12]. The potential involvement of alternative pathways has not been explored. Furthermore, LRG1 upregulation inhibits HIF‐1α hydroxylation, leading to its accumulation [14], which in turn drives pyroptosis and the release of inflammatory factors such as IL‐6 [41]. Continuously, IL‐6‐induced STAT3 activation upregulates the transcription of both LRG1 [11] and GSDME [46].

GSDME, a member of the gasdermin protein family, is among the most extensively studied GSDM proteins besides GSDMD [46]. Its activation occurs when caspase‐3 cleaves GSDME, enabling its N‐terminal fragment (N‐GSDME) to oligomerize and form plasma membrane pores, which leads to lytic cell death. Additionally, N‐GSDME can permeabilize the mitochondrial membrane to amplify caspase‐3 activation during apoptosis and inflammasome activation [47]. Consequently, GSDME responds to caspase‐3 activators by converting apoptotic signals into pyroptosis, a process further regulated by STAT3‐mediated transcriptional control of GSDME [46]. Previous studies have established that activation of the caspase‐3/GSDME pathway following CIRI triggers the release of numerous pro‐inflammatory factors, thereby exacerbating neuroinflammation and tissue damage. Despite the documented presence of GSDME in both the brain and spinal cord, its specific functional roles within the nervous system remain largely elusive [41, 48, 49]. According to previous studies, BAX, BCL2, Caspase‐3, and GSDME form a critical signaling axis within the mitochondrial apoptotic pathway, mediating the transition from “programmed apoptosis” to “inflammatory pyroptosis” [39]. The core mechanism involves the disruption of the balance between the pro‐apoptotic protein BAX and the anti‐apoptotic protein BCL2 in response to apoptotic stimuli [39]. Activated BAX integrates into the mitochondrial membrane and forms pores, leading to cytochrome c release and subsequent activation of Caspase‐3 [39]. In addition to executing classical apoptotic functions—such as cleaving cytoskeletal and DNA repair proteins—Caspase‐3 also cleaves GSDME when expressed in the cell [39]. This cleavage releases the N‐terminal pore‐forming domain of GSDME, which perforates the plasma membrane, resulting in cell swelling, rupture, and the release of intracellular contents [39]. As a result, what would otherwise be a “silent” apoptotic process transitions into an “inflammatory” pyroptotic event, significantly amplifying immune activation and anti‐tumor responses [39].

A key highlight of our research is the proposal that HBO‐PC can inhibit the shift from mitochondrial apoptosis to GSDME‐mediated pyroptosis triggered by CIRI. We have also delineated the key upstream and downstream components of the caspase‐3/GSDME inflammatory apoptosis pathway. Furthermore, we introduced the novel concept of a chain‐like vicious cycle underlying this pathological process and provided evidence that HBO‐PC exerts protective effects by disrupting this pathological cascade and suppressing inflammatory apoptosis. Our study thus innovatively reveals that HBO‐PC alleviates CIRI by inhibiting pyroptosis via disruption of the LRG1‐mediated HIF1α–GSDME–IL‐6–STAT3 positive feedback loop. These findings clarify the mechanistic basis for HBO‐PC–mediated neuroprotection and carry clinical significance by proposing a novel combined strategy integrating HBO‐PC with targeted molecular intervention. This approach may enhance the precision and reliability of HBO‐PC as a preventive measure for both primary and secondary stroke in clinical practice. Furthermore, we identified that the suppression of LRG1 by HBO‐PC induces a phenotypic shift in microglia from pro‐inflammatory to anti‐inflammatory, which acts as an additional mechanism synergistically ameliorating severe ischemia–reperfusion injury. However, our study is subject to several limitations and leaves certain questions open for future research. For instance, the regulatory interactions among the downstream proteins of LRG1 require further experimental validation. Additionally, HBO‐PC alone was observed to suppress LRG1 expression—an intriguing finding whose underlying mechanisms and biological implications merit more in‐depth investigation.

In this study, it is noteworthy that there were no statistically significant differences between the siLRG1 + MCAO/R group and the HBO‐PC + MCAO/R group in terms of cerebral infarct volume, neurological severity scores, behavioral performance or the expression of LRG1, inflammation‐related proteins and pyroptosis‐related proteins (Figure S3). These findings strongly suggest that the protective effects elicited by HBO‐PC and LRG1 knockdown are critically overlapping, further supporting the notion that inhibition of LRG1 may represent a core mechanism underlying HBO‐PC‐mediated neuroprotection. Meanwhile, the combined intervention (siLRG1 + HBO‐PC + MCAO/R) yielded greater benefits in alleviating cerebral ischemia–reperfusion injury, neuroinflammation and pyroptosis compared to LRG1 knockdown alone (siLRG1 + MCAO/R) (Figure 4). This observation indicates that HBO‐PC may also engage additional protective pathways independent of LRG1, such as scavenging reactive oxygen species, stabilizing mitochondrial membrane potential, suppressing NF‐кB‐mediated inflammation, and preserving blood–brain barrier integrity [40, 50, 51, 52]. Moreover, given that siRNA‐mediated knockdown rarely achieves complete depletion of the target protein, the enhanced effect of the combined treatment may also reflect further suppression of residual LRG1 by HBO‐PC, thereby producing an additive effect. Importantly, these auxiliary mechanisms do not diminish the central role of LRG1 as a key hub in the neuroprotective network activated by HBO‐PC. Rather, our data indicate that HBO‐PC primarily exerts its protective effects against post‐ischemic neuroinflammation and pyroptosis by suppressing LRG1 and disrupting the positive feedback loop it drives. This further underscores the therapeutic potential of targeting LRG1 in ischemic brain injury. However, the conclusions of this study still need to be further validated using LRG1 gene knockout mice and in vitro experiments.

Our experimental design primarily establishes an association between HBO‐PC and LRG1 downregulation along with their functional synergy, but falls short of demonstrating that HBO‐PC acts directly through LRG1 to confer neuroprotection. Although we believe our findings are meaningful as they provide the first link between LRG1 and the protective effects of HBO‐PC while also uncovering a potential role for LRG1 in modulating the HIF‐1α/GSDME/IL‐6/STAT3 signaling axis during CIRI. These observations offer important clues for future investigations into how HBO‐PC regulates LRG1. The study also raises whether LRG1 could function as a novel oxygen‐sensing molecule. It's an intriguing question worthy of further exploration. Future studies could explore this possibility through bioinformatic analysis to determine if LRG1 contains potential oxygen‐binding sites or redox‐sensitive disulfide bonds whose conformation might shift with changes in oxygen tension. In vitro experiments exposing LRG1‐expressing cells to various oxygen concentrations such as normoxia, hyperoxia, or hypoxia could help determine whether oxygen levels directly influence LRG1 expression, which would support the oxygen‐sensor hypothesis. Additionally, investigating whether HBO‐PC regulates LRG1 transcription by modulating the activity or DNA‐binding capacity of transcription factors like HIF‐1α or STAT3 could clarify the molecular bridge linking HBO‐PC to LRG1. In essence, our work provides preliminary in vivo evidence that may inform these future directions. We hope subsequent studies will dissect the direct or indirect relationship between HBO‐PC and LRG1 at the molecular level and further examine the possibility of LRG1 as an oxygen sensor. Such efforts could ultimately open new avenues for the prevention and treatment of ischemic stroke.

## 5 Conclusion

In summary, this study established a mouse model of MCAO/R and applied both HBO‐PC and siRNA interventions. Through a series of in vivo and molecular experiments, we demonstrated that HBO‐PC may reprogram neuroimmune metabolism through disruption of the LRG1‐HIF‐1α‐IL‐6‐STAT3 amplification loop, which attenuates pyroptosis and ischemia–reperfusion injury. Our findings identify LRG1 as the critical link whereby HBO‐PC suppresses HIF‐1α expression and confers neuroprotection. Furthermore, key signaling axes, including LRG1‐caspase‐3‐GSDME and IL‐6‐STAT3‐LRG1, were elucidated as central targets of HBO‐PC. These results enhance the credibility of HBO‐PC as a strategy for stroke prevention and provide a theoretical foundation for developing novel neuroprotective drugs targeting LRG1 or its associated pathways.

## Author Contributions

W.L. and J.L. were responsible for the construction of the MCAO model, sample collection, and data analysis. K.X. was responsible for molecular biology experiments. S.Y. conducted the behavior tests and was responsible for the data analysis. J.Y. was responsible for clarifying the manuscript content and word usage for an English language audience. D.G. conceived of the study, contributed to the formulation of overarching research goals, developed the proposal, and initiated the writing of the manuscript. All authors reviewed the manuscript.

## Funding

This work was supported by the National Key R&D Program of China (2022YFA1104300).

## Ethics Statement

All animal procedures were approved by the Ethics Committee of the 6th Medical Center, PLA General Hospital (No. JFJZYYDLYXZX, 2023–10) and reported in accordance with ARRIVE guidelines.

## Consent

Approval of article: all.

## Conflicts of Interest

The authors declare no conflicts of interest.

## Supporting information


**Figure S1:** Immunofluorescence co‐localization images of IL‐6 and IL‐10 with microglia and neurons, along with corresponding quantitative analysis statistical graphs.

**Figure S2:** Hyperbaric preconditioning attenuates microglial M1 polarization and neuronal pyroptosis via LRG1 suppression.

**Figure S3:** The comparison between the siLRG1 + MCAO/R group and HBO‐PC + MCAO/R group.

## Data Availability

The datasets used in this study are available from the corresponding author upon reasonable request.

## References

[1] GBD 2016 DALYs and HALE Collaborators , “Global, Regional, and National Disability‐Adjusted Life‐Years (DALYs) for 333 Diseases and Injuries and Healthy Life Expectancy (HALE) for 195 Countries and Territories, 1990–2016: A Systematic Analysis for the Global Burden of Disease Study 2016,” Lancet 390, no. 10100 (2017): 1260–1344, 10.1016/S0140-6736(17)32130-X. 28919118 PMC5605707

[2] T. Kalogeris , C. P. Baines , M. Krenz , and R. J. Korthuis , “Ischemia/Reperfusion. Comprehensive,” Physiology 7, no. 1 (2016): 113–170, 10.1002/cphy.c160006. PMC5648017 28135002

[3] J. Shi , W. Gao , and F. Shao , “Pyroptosis: Gasdermin‐Mediated Programmed Necrotic Cell Death,” Trends in Biochemical Sciences 42, no. 4 (2017): 245–254, 10.1016/j.tibs.2016.10.004. 27932073

[4] L. Poh , S. W. Kang , S. H. Baik , et al., “Evidence That NLRC4 Inflammasome Mediates Apoptotic and Pyroptotic Microglial Death Following Ischemic Stroke,” Brain, Behavior, and Immunity 75 (2019): 34–47, 10.1016/j.bbi.2018.09.001. 30195027

[5] S. O. Vasudevan , B. Behl , and V. A. Rathinam , “Pyroptosis‐Induced Inflammation and Tissue Damage,” Seminars in Immunology 69 (2023): 101781, 10.1016/j.smim.2023.101781. 37352727 PMC10598759

[6] J. Li , P. Xu , Y. Hong , et al., “Lipocalin‐2‐Mediated Astrocyte Pyroptosis Promotes Neuroinflammatory Injury via NLRP3 Inflammasome Activation in Cerebral Ischemia/Reperfusion Injury,” Journal of Neuroinflammation 20 (2023): 148, 10.1186/s12974-023-02819-5. 37353794 PMC10288712

[7] Q. Z. Tuo , S. T. Zhang , and P. Lei , “Mechanisms of Neuronal Cell Death in Ischemic Stroke and Their Therapeutic Implications,” Medicinal Research Reviews 42, no. 1 (2022): 259–305, 10.1002/med.21817. 33957000

[8] J. Zhang , N. Jiang , L. Zhang , C. Meng , J. Zhao , and J. Wu , “NLRP6 Expressed in Astrocytes Aggravates Neurons Injury After OGD/R Through Activating the Inflammasome and Inducing Pyroptosis,” International Immunopharmacology 80 (2020): 106183, 10.1016/j.intimp.2019.106183. 31927506

[9] P. An , J. Xie , S. Qiu , et al., “Hispidulin Exhibits Neuroprotective Activities Against Cerebral Ischemia Reperfusion Injury Through Suppressing NLRP3‐Mediated Pyroptosis,” Life Sciences 232 (2019): 116599, 10.1016/j.lfs.2019.116599. 31247210

[10] M. Zhao , W. Xian , W. Liu , D. Chen , S. Wang , and J. Cao , “Maresin1 Alleviates Neuroinflammation by Inhibiting Caspase‐3/GSDME‐Mediated Pyroptosis in Mice Cerebral Ischemia‐Reperfusion Model,” Journal of Stroke and Cerebrovascular Diseases 33, no. 8 (2024): 107789, 10.1016/j.jstrokecerebrovasdis.2024.107789. 38782167

[11] C. Camilli , A. E. Hoeh , G. D. Rossi , S. E. Moss , and J. Greenwood , “LRG1: An Emerging Player in Disease Pathogenesis,” Journal of Biomedical Science 29 (2022): 6, 10.1186/s12929-022-00790-6. 35062948 PMC8781713

[12] H. Urushima , M. Fujimoto , T. Mishima , et al., “Leucine‐Rich Alpha 2 Glycoprotein Promotes Th17 Differentiation and Collagen‐Induced Arthritis in Mice Through Enhancement of TGF‐β‐Smad2 Signaling in naïve Helper T Cells,” Arthritis Research & Therapy 19 (2017): 137, 10.1186/s13075-017-1349-2. 28615031 PMC5471956

[13] J. Jin , H. Sun , D. Liu , et al., “LRG1 Promotes Apoptosis and Autophagy Through the TGFβ‐smad1/5 Signaling Pathway to Exacerbate Ischemia/Reperfusion Injury,” Neuroscience 413 (2019): 123–134, 10.1016/j.neuroscience.2019.06.008. 31220542

[14] J. Feng , J. Zhan , and S. Ma , “LRG1 Promotes Hypoxia‐Induced Cardiomyocyte Apoptosis and Autophagy by Regulating Hypoxia‐Inducible Factor‐1α,” Bioengineered 12, no. 1 (2021): 8897–8907, 10.1080/21655979.2021.1988368. 34643170 PMC8806971

[15] A. Greco , G. Occhipinti , D. Giacoppo , et al., “Antithrombotic Therapy for Primary and Secondary Prevention of Ischemic Stroke: JACC State‐of‐the‐Art Review,” Journal of the American College of Cardiology 82, no. 15 (2023): 1538–1557, 10.1016/j.jacc.2023.07.025. 37793752

[16] C. Flach , W. Muruet , C. D. Wolfe , A. Bhalla , and A. Douiri , “Risk and Secondary Prevention of Stroke Recurrence: A Population‐Base Cohort Study,” Stroke 51, no. 8 (2020): 2435–2444, 10.1161/STROKEAHA.120.028992. 32646337 PMC7382537

[17] Z. X. Gao , J. Rao , and Y. H. Li , “Hyperbaric Oxygen Preconditioning Improves Postoperative Cognitive Dysfunction by Reducing Oxidant Stress and Inflammation,” Neural Regeneration Research 12, no. 2 (2017): 329–336, 10.4103/1673-5374.200816. 28400818 PMC5361520

[18] Z. J. Yang , Y. Xie , G. M. Bosco , C. Chen , and E. M. Camporesi , “Hyperbaric Oxygenation Alleviates MCAO‐Induced Brain Injury and Reduces Hydroxyl Radical Formation and Glutamate Release,” European Journal of Applied Physiology 108, no. 3 (2010): 513–522, 10.1007/s00421-009-1229-9. 19851780

[19] Z. Fang , Y. Feng , Y. Li , et al., “Neuroprotective Autophagic Flux Induced by Hyperbaric Oxygen Preconditioning Is Mediated by Cystatin C,” Neuroscience Bulletin 35, no. 2 (2018): 336–346, 10.1007/s12264-018-0313-8. 30519802 PMC6426805

[20] Q. Li , J. Li , L. Zhang , B. Wang , and L. Xiong , “Preconditioning With Hyperbaric Oxygen Induces Tolerance Against Oxidative Injury via Increased Expression of Heme Oxygenase‐1 in Primary Cultured Spinal Cord Neurons,” Life Sciences 80, no. 12 (2007): 1087–1093, 10.1016/j.lfs.2006.11.043. 17291539

[21] G. J. Gu , Y. P. Li , Z. Y. Peng , et al., “Mechanism of Ischemic Tolerance Induced by Hyperbaric Oxygen Preconditioning Involves Upregulation of Hypoxia‐Inducible Factor‐1alpha and Erythropoietin in Rats,” Journal of Applied Physiology 104, no. 4 (2008): 1185–1191, 10.1152/japplphysiol.00323.2007. 18174394

[22] R. P. Ostrowski , V. Jadhav , W. Chen , and J. H. Zhang , “Reduced Matrix Metalloproteinase‐9 Activity and Cell Death After Global Ischemia in the Brain Preconditioned With Hyperbaric Oxygen,” Acta Neurochirurgica Supplement 106 (2010): 47–49, 10.1007/978-3-211-98811-4_7. 19812919

[23] Y. Soejima , Q. Hu , P. R. Krafft , M. Fujii , J. Tang , and J. H. Zhang , “Hyperbaric Oxygen Preconditioning Attenuates Hyperglycemia‐Enhanced Hemorrhagic Transformation by Inhibiting Matrix Metalloproteinases in Focal Cerebral Ischemia in Rats,” Experimental Neurology 247 (2013): 737–743, 10.1016/j.expneurol.2013.03.019. 23537951 PMC3742563

[24] W. Li , J. Lan , M. Wei , et al., “Normobaric Hyperoxia Combined With Endovascular Treatment for Acute Ischaemic Stroke in China (OPENS‐2 Trial): A Multicentre, Randomised, Single‐Blind, Sham‐Controlled Trial,” Lancet 405, no. 10477 (2025): 486–497, 10.1016/S0140-6736(24)02809-5. 39922675

[25] Z. Shi , K. Zhang , H. Zhou , et al., “Increased miR‐34c Mediates Synaptic Deficits by Targeting Synaptotagmin 1 Through ROS‐JNK‐p53 Pathway in Alzheimer's Disease,” Aging Cell 19, no. 3 (2020): e13125, 10.1111/acel.13125. 32092796 PMC7059146

[26] Z. Ruan , G. Cao , Y. Qian , et al., “Single‐Cell RNA Sequencing Unveils Lrg1's Role in Cerebral Ischemia–Reperfusion Injury by Modulating Various Cells,” Journal of Neuroinflammation 20 (2023): 285, 10.1186/s12974-023-02941-4. 38037097 PMC10687904

[27] H. Nie , L. Xiong , N. Lao , S. Chen , N. Xu , and Z. Zhu , “Hyperbaric Oxygen Preconditioning Induces Tolerance Against Spinal Cord Ischemia by Upregulation of Antioxidant Enzymes in Rabbits,” Journal of Cerebral Blood Flow and Metabolism 26, no. 5 (2006): 666–674, 10.1038/sj.jcbfm.9600221. 16136055

[28] Q. Wen , Y. Wang , Q. Pan , et al., “MicroRNA‐155‐5p Promotes Neuroinflammation and Central Sensitization via Inhibiting SIRT1 in a Nitroglycerin‐Induced Chronic Migraine Mouse Model,” Journal of Neuroinflammation 18, no. 1 (2021): 287, 10.1186/s12974-021-02342-5. 34893074 PMC8665643

[29] Y. Qian , L. Yang , J. Chen , et al., “SRGN Amplifies Microglia‐Mediated Neuroinflammation and Exacerbates Ischemic Brain Injury,” Journal of Neuroinflammation 21, no. 1 (2024): 35, 10.1186/s12974-024-03026-6. 38287411 PMC10826034

[30] R. D'Hooge and P. P. De Deyn , “Applications of the Morris Water Maze in the Study of Learning and Memory. Brain Research,” Brain Research Reviews 36, no. 1 (2001): 60–90, 10.1016/s0165-0173(01)00067-4. 11516773

[31] J. Nunez , “Morris Water Maze Experiment,” Journal of Visualized Experiments 19 (2008): 897, 10.3791/897. PMC2872979 19066539

[32] J. Chen , Y. Li , L. Wang , et al., “Therapeutic Benefit of Intravenous Administration of Bone Marrow Stromal Cells After Cerebral Ischemia in Rats,” Stroke 32, no. 4 (2001): 1005–1011, 10.1161/01.str.32.4.1005. 11283404

[33] R. J. Carter , J. Morton , and S. B. Dunnett , Motor Coordination and Balance in Rodents, In Current Protocols in Neuroscience, vol. 15, 8.12.1–8.12.14 (2001). John Wiley & Sons. 10.1002/0471142301.ns0812s15. 18428540

[34] G. Wu , D. W. McBride , and J. H. Zhang , “Axl Activation Attenuates Neuroinflammation by Inhibiting the TLR/TRAF/NF‐кB Pathway After MCAO in Rats,” Neurobiology of Disease 110 (2018): 59–67, 10.1016/j.nbd.2017.11.009. PMC5748011 29196212

[35] J. Chen , Y. Li , and M. Chopp , “Intracerebral Transplantation of Bone Marrow With BDNF After MCAO in Rat,” Neuropharmacology 39, no. 5 (2000): 711–716, 10.1016/s0028-3908(00)00006-x. 10699437

[36] L. Zhang , J. Chen , Y. Li , Z. G. Zhang , and M. Chopp , “Quantitative Measurement of Motor and Somatosensory Impairments After Mild (30 Min) and Severe (2 h) Transient Middle Cerebral Artery Occlusion in Rats,” Journal of the Neurological Sciences 174, no. 2 (2000): 141–146, 10.1016/s0022-510x(00)00268-9. 10727700

[37] V. Bouet , M. Boulouard , J. Toutain , et al., “The Adhesive Removal Test: A Sensitive Method to Assess Sensorimotor Deficits in Mice,” Nature Protocols 4, no. 10 (2009): 1560–1564, 10.1038/nprot.2009.125. 19798088

[38] P. Xu , Q. Liu , Y. Xie , et al., “Breast Cancer Susceptibility Protein 1 (BRCA1) Rescues Neurons From Cerebral Ischemia/Reperfusion Injury Through NRF2‐Mediated Antioxidant Pathway,” Redox Biology 18 (2018): 158–172, 10.1016/j.redox.2018.06.012. 30014904 PMC6068089

[39] Y. Wang , Y. Cui , Z. Liu , et al., “Acevaltrate Overcomes Myeloma Resistance to Bortezomib via Pyroptosis by Promoting BAX Translocalization to Mitochondria,” European Journal of Pharmacology 996 (2025): 177572, 10.1016/j.ejphar.2025.177572. 40180268

[40] S. D. Wang , Y. Y. Fu , X. Y. Han , et al., “Hyperbaric Oxygen Preconditioning Protects Against Cerebral Ischemia/Reperfusion Injury by Inhibiting Mitochondrial Apoptosis and Energy Metabolism Disturbance,” Neurochemical Research 46, no. 4 (2021): 866–877, 10.1007/s11064-020-03219-4. 33453006

[41] Z. Xueqiang , Z. Yu , L. Wendong , F. Shuying , and H. Jie , “Salidroside Alleviates Renal Ischemia‐Reperfusion Injury by Inhibiting Macrophage Pyroptosis Through HIF Signaling,” Inflammation 48 (2025): 4318–4329, 10.1007/s10753-025-02328-y. 40591075 PMC12722482

[42] Y. Luo , H. Tang , H. Li , R. Zhao , Q. Huang , and J. Liu , “Recent Advances in the Development of Neuroprotective Agents and Therapeutic Targets in the Treatment of Cerebral Ischemia,” European Journal of Medicinal Chemistry 162 (2019): 132–146, 10.1016/j.ejmech.2018.11.014. 30445263

[43] L. Sun , K. Xie , C. Zhang , R. Song , and H. Zhang , “Hyperbaric Oxygen Preconditioning Attenuates Postoperative Cognitive Impairment in Aged Rats,” Neuroreport 25, no. 9 (2014): 718–724, 10.1097/WNR.0000000000000181. 24870985

[44] S. L. Pek , S. Tavintharan , X. Wang , et al., “Elevation of a Novel Angiogenic Factor, Leucine‐Rich‐α2‐Glycoprotein (LRG1), is Associated With Arterial Stiffness, Endothelial Dysfunction, and Peripheral Arterial Disease in Patients With Type 2 Diabetes,” Journal of Clinical Endocrinology and Metabolism 100, no. 4 (2015): 1586–1593, 10.1210/jc.2014-3855. 25636050

[45] H. Meng , Y. Song , J. Zhu , et al., “LRG1 Promotes Angiogenesis Through Upregulating the TGF‐ß1 Pathway in Ischemic Rat Brain,” Molecular Medicine Reports 14, no. 6 (2016): 5535–5543, 10.3892/mmr.2016.5925. 27840991 PMC5355675

[46] Y. Wei , B. Lan , T. Zheng , et al., “GSDME‐Mediated Pyroptosis Promotes the Progression and Associated Inflammation of Atherosclerosis,” Nature Communications 14, no. 1 (2023): 929, 10.1038/s41467-023-36614-w. PMC9938904 36807553

[47] C. Rogers , D. A. Erkes , A. Nardone , A. E. Aplin , T. Fernandes‐Alnemri , and E. S. Alnemri , “Gasdermin Pores Permeabilize Mitochondria to Augment Caspase‐3 Activation During Apoptosis and Inflammasome Activation,” Nature Communications 10, no. 1 (2019): 1689, 10.1038/s41467-019-09397-2. PMC6459836 30976076

[48] W. Xia , Y. Li , M. Wu , et al., “Gasdermin E Deficiency Attenuates Acute Kidney Injury by Inhibiting Pyroptosis and Inflammation,” Cell Death & Disease 12, no. 2 (2021): 139, 10.1038/s41419-021-03431-2. 33542198 PMC7862699

[49] Y. Li , G. Xin , S. Li , et al., “PD‐L1 Regulates Platelet Activation and Thrombosis via Caspase‐3/GSDME Pathway,” Frontiers in Pharmacology 13 (2022): 921414, 10.3389/fphar.2022.921414. 35784685 PMC9240427

[50] X. Wu , J. You , X. Chen , et al., “An Overview of Hyperbaric Oxygen Preconditioning Against Ischemic Stroke,” Metabolic Brain Disease 38, no. 3 (2023): 855–872, 10.1007/s11011-023-01165-y. 36729260 PMC10106353

[51] S. Kovacevic , N. Mitovic , P. Brkic , et al., “Hyperbaric Oxygenation: Can It be a Novel Supportive Method in Acute Kidney Injury? Data Obtained From Experimental Studies,” Cells 13, no. 13 (2024): 1119, 10.3390/cells13131119. 38994971 PMC11240597

[52] J. You , X. Chen , M. Zhou , H. Ma , Q. Liu , and C. Huang , “Hyperbaric Oxygen Preconditioning for Prevention of Acute High‐Altitude Diseases: Fact or Fiction?,” Frontiers in Physiology 14 (2023): 1019103, 10.3389/fphys.2023.1019103. 36760528 PMC9905844

