# Supplementary material for: HBO‐PC Reprograms Neuroimmune Metabolism Through Disruption of the LRG1‐HIF‐1α‐IL‐6‐STAT3 Amplification Loop Attenuates Pyroptosis and Ischemia–Reperfusion Injury
Source: CNS Neurosci Ther. 2026 Apr 29;32(5):e70907. doi: 10.1002/cns.70907 (PMC13127231; doi:10.1002/cns.70907)
Supplement: Supplementary file 1 — Figure S1: Immunofluorescence co‐localization images of IL‐6 and IL‐10 with microglia and neurons, along with corresponding quantitative analysis statistical graphs. [file CNS-32-e70907-s003.docx]

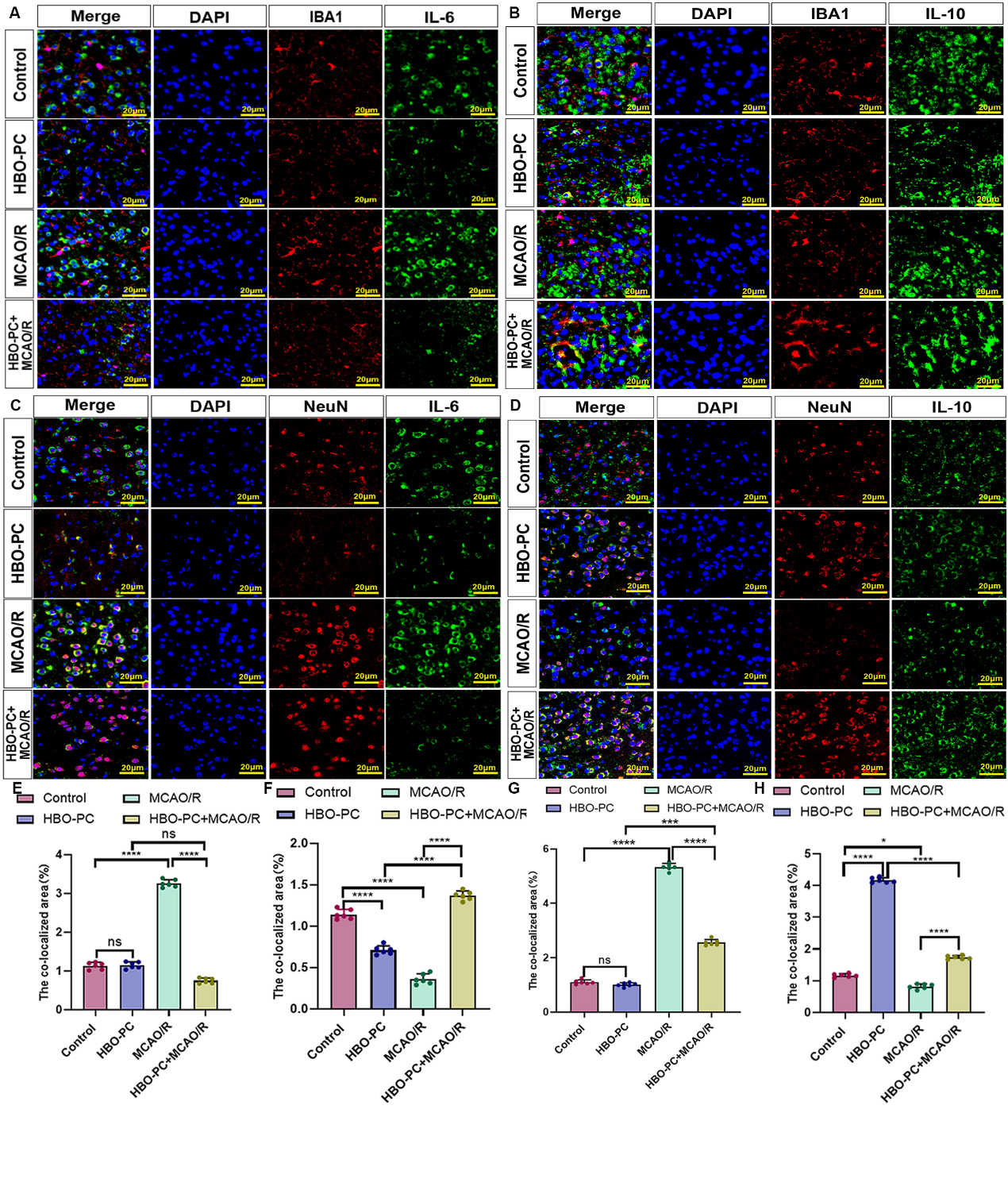


**Supplementary Figure1. Immunofluorescence co-localization images of IL-6 and IL-10 with microglia and neurons, along with corresponding quantitative analysis statistical graphs.**

1. Double immunofluorescence analysis was performed using antibodies against IL-6 (green) and IBA1 (red) in brain sections. Nuclei were fluorescently labeled with DAPI (blue). Merged images show increased colocalization of IL-6 and IBA-1 increased co- localization after cerebral ischemia-reperfusion injury (n = 6 rats per group). Scale bars, 20μm.
2. Double immunofluorescence analysis was performed using antibodies against IL-10(green) and IBA1 (red) in brain sections. Nuclei were fluorescently labeled with DAPI (blue). Merged images show increased colocalization of IL-10 and IBA-1 increased co- localization after cerebral ischemia-reperfusion injury (n = 6 rats per group). Scale bars, 20μm.
3. Double immunofluorescence analysis was performed using antibodies against IL-6(green) and NEUN (red) in brain sections. Nuclei were fluorescently labeled with DAPI (blue). Merged images show increased colocalization of IL-6 and NEUN increased co- localization after cerebral ischemia-reperfusion injury (n = 6 rats per group). Scale bars, 20μm.
4. Double immunofluorescence analysis was performed using antibodies against IL-10(green) and NEUN (red) in brain sections. Nuclei were fluorescently labeled with DAPI (blue). Merged images show increased colocalization of IL-10 and NEUN increased co- localization after cerebral ischemia-reperfusion injury (n = 6 rats per group). Scale bars, 20μm.
5. Bar chart showing the total area of co-localized expression of IL-6 and IBA-1 (n = 6 in each group). ****, p＜0.0001; ns, there was no statistically significant difference between the two groups.
6. Bar chart showing the total area of co-localized expression of IL-10 and IBA-1 (n = 6 in each group). ****, p＜0.0001.
7. Bar chart showing the total area of co-localized expression of IL-6 and NEUN (n = 6 in each group). ****, p＜0.0001; ***, p<0.001; ns, there was no statistically significant difference between the two groups.
8. Bar chart showing the total area of co-localized expression of IL-10 and NEUN (n = 6 in each group). *, p<0.05; ****, p＜0.0001.
