# Supplementary material for: HBO‐PC Reprograms Neuroimmune Metabolism Through Disruption of the LRG1‐HIF‐1α‐IL‐6‐STAT3 Amplification Loop Attenuates Pyroptosis and Ischemia–Reperfusion Injury
Source: CNS Neurosci Ther. 2026 Apr 29;32(5):e70907. doi: 10.1002/cns.70907 (PMC13127231; doi:10.1002/cns.70907)
Supplement: Supplementary file 2 — Figure S2: Hyperbaric preconditioning attenuates microglial M1 polarization and neuronal pyroptosis via LRG1 suppression. [file CNS-32-e70907-s001.docx]

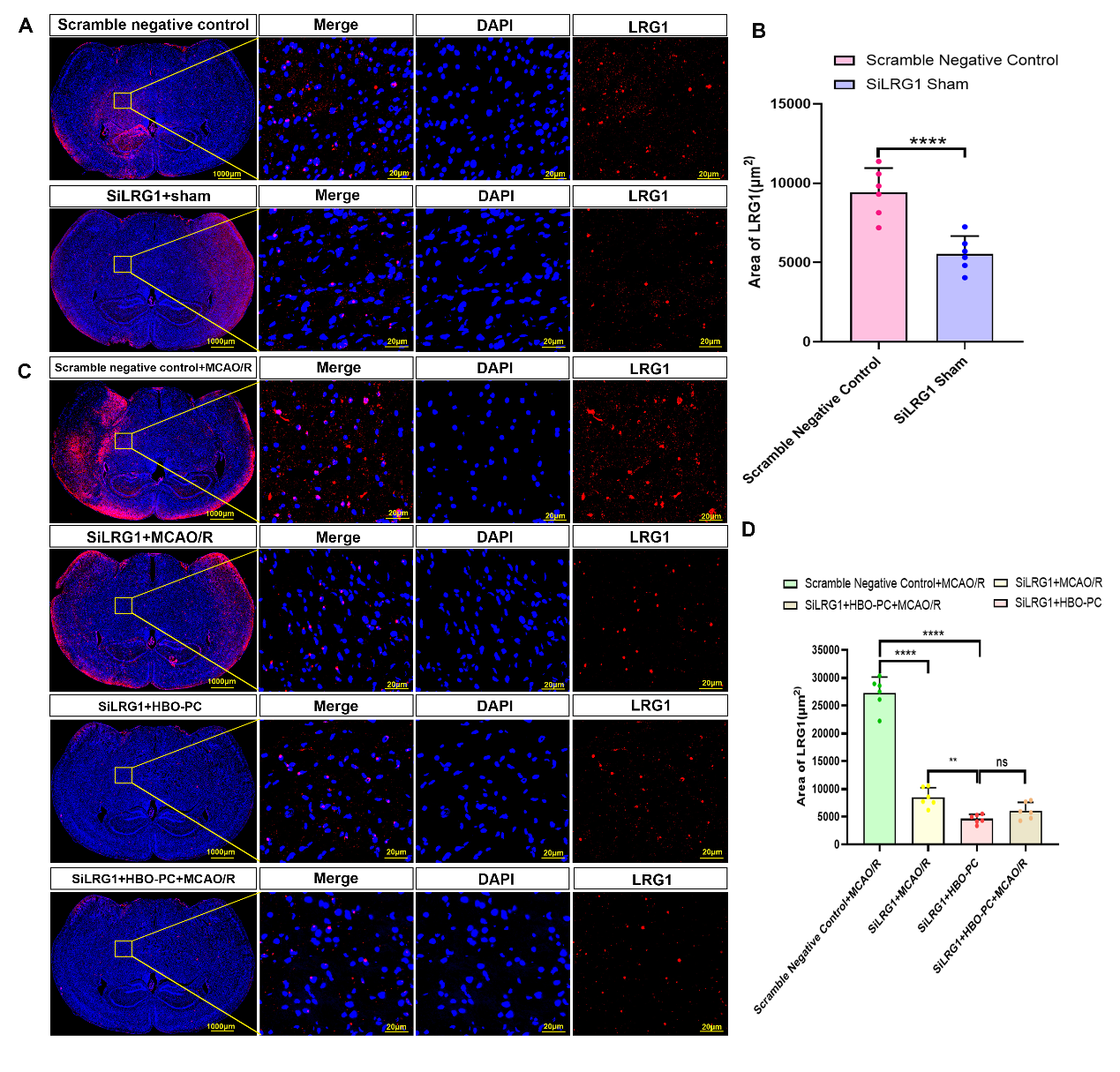


**Supplementary Figure 2. Hyperbaric preconditioning attenuates microglial M1 polarization and neuronal pyroptosis via LRG1 suppression**

1. Immunofluorescence staining was performed with LRG1 antibody (red) in brain sections. Nuclear fluorescent labeling with DAPI (blue) (n = 6 rats per group). Scale bars, 1000 μm and 20 μm.
2. The mean area of LRG1(n = 6 rats per group). ****p < 0.00001.
3. Immunofluorescence staining was performed with LRG1 antibody (red) in brain sections. Nuclear fluorescent labeling with DAPI (blue) (n = 6 rats per group). Scale bars, 1000 μm and 20 μm.
4. The mean area of LRG1(n = 6 rats per group). ****, p < 0.00001; **, p<0.01; ns, there was no statistically significant difference between the two groups.
