# Supplementary material for: HBO‐PC Reprograms Neuroimmune Metabolism Through Disruption of the LRG1‐HIF‐1α‐IL‐6‐STAT3 Amplification Loop Attenuates Pyroptosis and Ischemia–Reperfusion Injury
Source: CNS Neurosci Ther. 2026 Apr 29;32(5):e70907. doi: 10.1002/cns.70907 (PMC13127231; doi:10.1002/cns.70907)
Supplement: Supplementary file 3 — Figure S3: The comparison between the siLRG1 + MCAO/R group and HBO‐PC + MCAO/R group. [file CNS-32-e70907-s002.docx]

**
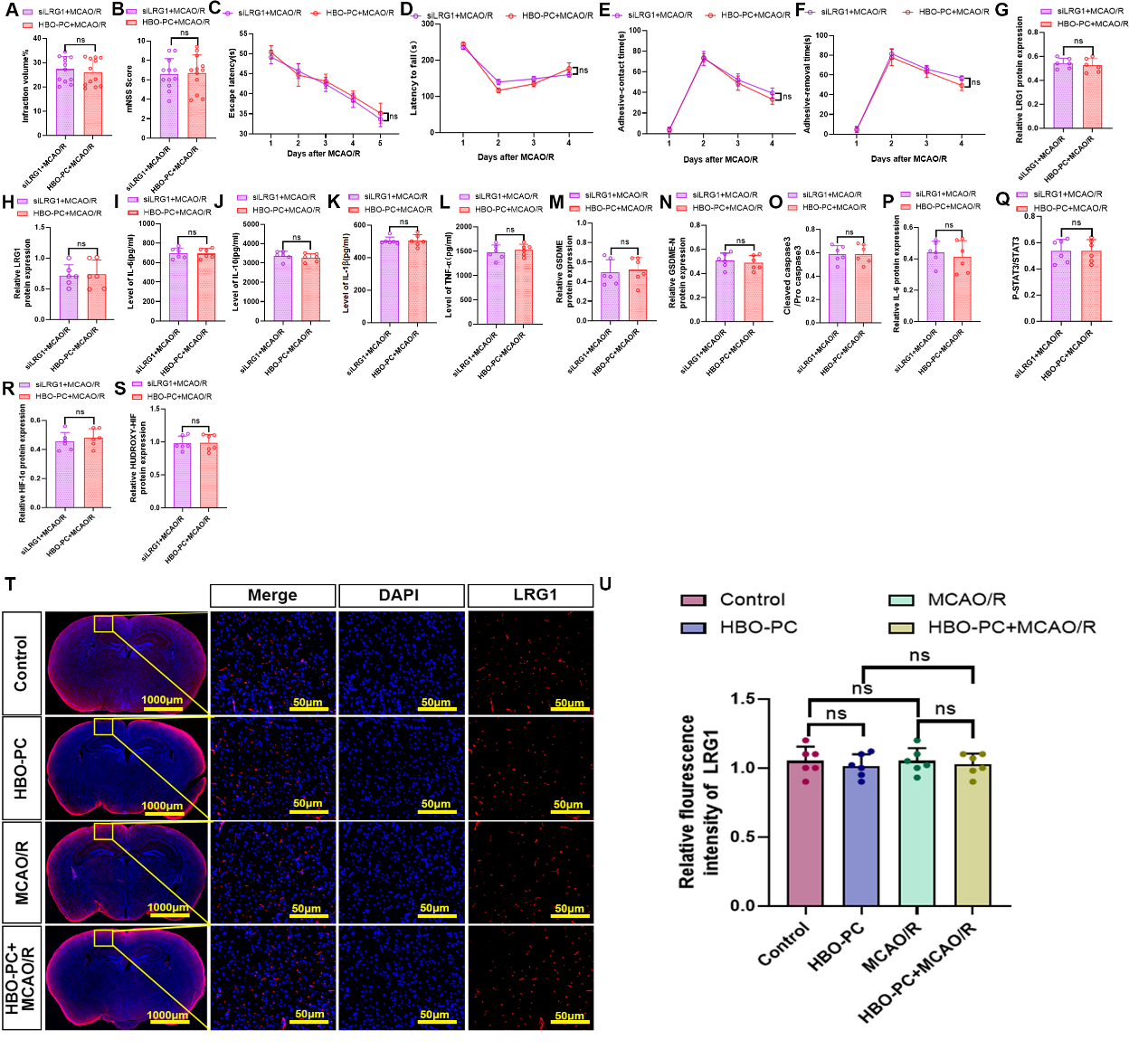
Supplementary Figure 3. The comparison between the siLRG1 +MCAO/R group and HBO-PC+MCAO/R group.**

1. Volume statistics chart of cerebral infarction of of brain sections (n=12 mice per group). ns, there was no statistically significant difference between the two groups.
2. Modified neurological severity score (mNSS) test (n=12 mice per group). ns, there was no statistically significant difference between the two groups.
3. The mean escape latency in the morris water maze (n=12 mice per group). ns, there was no statistically significant difference between the two groups.
4. Latency to fall in the rotarod test (n=12 mice per group). ns, there was no statistically significant difference between the two groups.
5. Time required to feel the adhesive tapes during adhesive contact test (contact time) (n=12 mice per group). ns, there was no statistically significant difference between the two groups.
6. Time required to feel the adhesive tapes during adhesive removal test (removal time) (n=12 mice per group). ns, there was no statistically significant difference between the two groups.
7. The statistical chart of relative protein expression of LRG1 (fold). ns, there was no statistically significant difference between the two groups.
8. The relative fluorescence of LRG1(n = 6 rats per group). ns, there was no statistically significant difference between the two groups.
9. ELISA of IL-6. The bar chart of IL-6 concentration levels, ns, there was no statistically significant difference between the two groups.
10. ELISA of IL-10. The bar chart of IL-10 concentration levels. ns, there was no statistically significant difference between the two groups.
11. ELISA of IL-1ß. The bar chart of IL-1ß concentration levels. ns, there was no statistically significant difference between the two groups.
12. ELISA of TNF-α. The bar chart of TNF-α concentration levels. ns, there was no statistically significant difference between the two groups.
13. The statistical chart of relative protein expression of GSDME (fold). ns, there was no statistically significant difference between the two groups.
14. The statistical chart of relative protein expression of GSDME-N (fold). ns, there was no statistically significant difference between the two groups.
15. The Cleaved caspase3/ Pro caspase3(fold). ns, there was no statistically significant difference between the two groups.
16. The statistical chart of relative protein expression of IL-6 (fold) (n = 6 rats per group). ns, there was no statistically significant difference between the two groups.
17. The P-STAT3/STAT3 (fold) (n = 6 rats per group). ns, there was no statistically significant difference between the two groups.
18. The statistical chart of relative protein expression of HIF-1α (fold) (n = 6 rats per group). ns, there was no statistically significant difference between the two groups.
19. The statistical chart of relative protein expression of HUDROXY-HIF n (fold) (n = 6 rats per group). ns, there was no statistically significant difference between the two groups.
20. Immunofluorescence staining was performed with LRG1 antibody (red) in brain sections. Nuclear fluorescent labeling with DAPI (blue) (n = 6 rats per group). Scale bars, 1000 μm and 50 μm.
21. The relative fluorescence of LRG1(n = 6 rats per group). ns, there was no statistically significant difference between the two groups.
